# Replication study of susceptibility variants associated with allergic rhinitis and allergy in Han Chinese

**DOI:** 10.1186/s13223-020-0411-9

**Published:** 2020-02-11

**Authors:** Yunbo Gao, Jingyun Li, Yuan Zhang, Luo Zhang

**Affiliations:** 1 Department of Otolaryngology Head and Neck Surgery, Beijing TongRen Hospital, Capital Medical University, Beijing, 100730 People’s Republic of China; 2 Beijing Key Laboratory of Nasal Diseases, Beijing Institute of Otolaryngology, No. 17, HouGouHuTong, DongCheng District, Beijing, 100005 People’s Republic of China; 3 Department of Allergy, Beijing TongRen Hospital, Capital Medical University, Beijing, 100730 People’s Republic of China

**Keywords:** Allergic rhinitis, Single nucleotide polymorphism, Replication

## Abstract

**Background:**

Allergic rhinitis (AR) is believed to be a complex genetic disease. The last decade has been marked by the publication of more than 20 genome-wide association studies (GWASs) of AR and associated allergic phenotypes and allergic diseases, which have shown allergic diseases and traits to share a large number of genetic susceptibility loci. The aim of present study was therefore to investigate the highly replicated allergy related genes and variants as candidates for AR in Han Chinese subjects.

**Methods:**

A total of 762 AR patients and 760 control subjects were recruited, and a total of 58 susceptible variants previously reported to be associated with allergic traits were choose for replication.

**Results:**

Logistic regression analyses revealed that in the co-dominant-effect model as assessed by the AIC, compared with wild-type carriers, significant AR risk were associated with rs9865818 in LPP (*P* = 0.029, OR = 1.469 for GG vs. AA); rs6554809 in DNAH5 (*P* = 0.000, OR = 1.597 for TC vs. CC); rs1438673 in WDR36-CAMK4 loci (*P* = 0.037, OR = 1.396 for CC vs.TT), rs7775228 in HLA region (*P* = 0.000, OR = 1.589 for TC vs.TT), rs7203459 in CLEC16A (*P* = 0.025, OR = 0.731 for TC vs. TT).

**Conclusion:**

We replicated Han Chinese AR-specific susceptibility loci in LPP, DNAH5, HLA, CLEC16A and WDR36-CAMK4. Further understanding the molecular mechanisms underlying these associations may provide new insights into the etiology of allergic disease.

## Background

Allergic rhinitis (AR) is a major chronic respiratory disease induced by an immunoglobulin E (IgE)‐mediated reaction in allergen‐sensitized subjects. It is believed that AR and the comorbid conditions including allergic asthma, eczema, or any other allergic disease, are complex genetic diseases resulting from the effect of both multiple genetic and interacting environmental factors on their pathophysiology. Moreover, Barnes [1] has proposed that as allergic diseases such as asthma, AR, and atopic dermatitis share common systemic characteristics [e.g. high total and/or specific IgE (sIgE)], then it was reasonable that a number of susceptibility genes could contribute to the allergic process regardless of the specific clinical phenotype.

The last decade has been marked by the publication of more than 20 genome-wide association studies (GWASs) of AR, atopy and the associated allergic phenotypes and allergic diseases and traits [2–7]. Andiappan et al. [8] first carried out GWAS strategy to investigate the associations of novel genetic variants with AR in a cohort of ethnic Chinese in Singapore. We previously performed the independent replication of these AR susceptibility genes in a Han Chinese population [9]. Recently, four large-scale, meta-based GWASs were of interest to identify the genetic variants that affect the susceptibility to AR and allergy. Ramasamy et al. [10] reported 3 genome-wide and 12 suggestively significant loci using existing GWAS data as well as 3 loci using a candidate gene approach associated with self-reported seasonal AR and grass sensitization in four large European cohorts. Bønnelykke [11] and colleagues found 10 genome-wide associated loci for allergic sensitization in a GWAS meta-analysis of subjects of European descent. Hinds et al. [12] identified 16 genome-wide and 6 suggestively significant loci in a GWAS meta-analysis for shared effects across pollen, dust mite and cat allergies also in European cohorts. Recently a study integrated GWAS, coexpression network and expression SNP analysis and reported 5 genome-wide significant loci for AR in ethnically diverse North American individuals [13]. Besides, a genome-wide linkage scan in 295 families of French Epidemiological Study showed strong evidence of linkage of NFIA locus to the combined asthma plus AR phenotype [14]. Bønnelykke et al*. [*15] reported 5 genome-wide significant loci in a GWAS of early childhood asthma with severe exacerbations. Of these, 4 loci (GSDMB, IL33, RAD50 and IL1R1) were hot spot as allergic traits susceptibility loci with larger effect sizes, as well as CDHR3 was a new susceptibility gene for asthma, which also was found to be associated with chronic rhinosinusitis [15], suggesting their potential relevance to allergic rhinitis. Together 6 studies reported a set of 58 genome-wide significant or suggestively significant SNPs in 55 loci as associated with AR and allergy phenotypes. The functional implications of above mentioned loci were evaluated using multi-gene-list meta-analysis at the level of pathways or protein complexes with Metascape website. Cytokines and immunity related pathways, such as cytokine production, Th17 cell differential and regulation of cytokine secretion, were significantly enriched and shared among GWASs (Additional file 1: Figure S1). However, few of studies have been convincingly and highly replicated especially in Chinese [5, 16]. The aim of the present study is to investigate the highly replicated allergy related genes and variants as candidates for AR in Han Chinese.

## Materials and methods

### Study subjects

The study protocol was approved by the Ethics Committee of Beijing TongRen Hospital and performed in accordance with the guidelines of the World Medical Association’s Declaration of Helsinki. All subjects involved were adults (≥ 18 years) of Han Chinese ethnic origin from the Beijing region, China, and provided written informed consent prior to entry in the study.

Two rhinology specialists (Y. Z. and L. Z.) were responsible for the screening the study subjects in outpatient clinic of Allergy department at Beijing TongRen Hospital, during the study period from February 2010 to February 2011. Finally, a total of 762 consecutive adult subjects with physician-diagnosed clinical AR were recruited. The flow diagram of recruitment was shown in Additional file 1: Figure S2. The diagnosis of AR fulfilled all criteria of the Allergic Rhinitis and its Impact on Asthma (ARIA) guidelines [17], including (i) presence of persistent or discontinuous symptoms of anterior rhinorrhoea, continuous sneezing, nasal obstruction and itching, (ii) demonstration of a pale and edematous nasal mucosa, nasal discharge and swollen inferior turbinate by nasal endoscopy, and (iii) positive serum antigen sIgE, measured by the ImmunoCAP 100 system (Pharmacia, Uppsala, Sweden). A diagnosis of AR was further confirmed by the presence of symptoms induced by exposure to allergen shown to produce positive serum allergen sIgE response. The tested allergens included house dust mite (HDM) (*Der f* and *Der p*); seasonal grass pollens (Giant Ragweed; Mugwort; Lamb’s quarters; Humulus; Chenopodium album); animal hair (dog and cat); molds (indoor and outdoor mustiness or floricultural environment) and cockroach. Subjects were also considered to be sensitized to allergens when the serum sIgE was greater than 0.35 kU/l. AR subjects with (i) co-morbid asthma, eczema, or any other allergic disease; (ii) hypertension, diabetes or other chronic diseases; or (iii) tumor in the nasal cavity or any other inflammatory nasal disease were excluded. The diagnosis of asthma was confirmed by a physician according to Global Initiative for Asthma (GINA) guidelines [18].

A total of 760 adult healthy control volunteers were also recruited during the study period. For matching with AR groups, health control group had similar age distribution, gender ratio, and also Han Chinese ethnic origin from the Beijing region for an ethnically similar local population to determine background population allele frequencies. None of the control subjects had a history of allergic or any nasal disease, nor demonstrated any abnormal clinical features in the nasal cavity or positive serum sIgE by Phadiatop (Pharmacia, Uppsala, Sweden) testing.

### Extraction of genomic DNA

A 2-ml volume of venous blood samples from each participant was taken in citrate-anticoagulated glass tubes, and were frozen at − 40 °C. Total genomic DNA of the leucocyte was extracted from 1 ml of peripheral blood using the Whole Blood DNA Extraction Kit (Tiangen Biotech, Co., Ltd, Beijing, China), according to the manufacturer’s instructions. Genomic DNA extracted was dissolved in 0.1× TE buffer (10 mMTris-1 mM EDTA, pH 8.0) and stored at − 20 °C.

### Replication SNP selection

58 susceptible single nucleotide polymorphisms (SNPs) in 55 gene/regions previously reported as highly associated with allergic traits [11–13, 15] were chosen for replication. The detailed information of the selected candidate SNPs were summarized in Additional file 1: Table S1.

### SNP genotyping assays

SNPs were typed using iPLEX chemistry on a matrix-assisted laser desorption/ionization time-of-flight mass spectrometer (MALDI-TOF–MS, named as MassARRAY system, manufactured by Sequenom, Inc.) [19]. In brief, (i) Multiplex PCR amplification: PCR reactions were carried out in standard 384-well plates in 5 μl per reaction with 10 ng of genomic DNA, 0.5 units of Taq polymerase (HotStarTaq, Qiagen), 500 μmol of each deoxynucleotide triphosphate (dNTP), and 100 nmol of each PCR primer. PCR thermal cycling was carried out in an ABI-9700 instrument for 15 min at 94 °C, followed by 45 cycles of 20 s at 94 °C, 30 s at 56 °C, and 60 s at 72 °C. PCR products were electrophoresed on 2.0% agarose. (ii) Removing residual primers and dNTPs: After PCR reaction, 2 μl containing 0.3 units of Shrimp Alkaline Phosphatase was added, and the reaction was incubated at 37 °C for 20 min followed by inactivation for 5 min at 85 °C. (iii) PCR with single base extension: After adjusting the concentrations of extension primers to equilibrate signal-to-noise ratios, the post-PCR primer extension reaction of the iPLEX Gold Kits (Sequenom, Inc.) assay was done in a final 9 μl volume extension reaction containing 0.2 μl (100 μmol) of termination mix, 0.04 μl containing 0.05 units of DNA polymerase (Sequenom, Inc.), and 625 to 1250 nmol/l extension primers. A 200-short-cycle program was used for the iPLEX reaction: initial denaturation was for 30 s at 94 °C followed by 5 s at 94 °C and five cycles of 5 s at 52 °C and 5 s at 80 °C. An additional 40 annealing and extension cycles were then looped back to 5 s at 94 °C, five cycles of 5 s at 52 °C and 5 s at 80 °C. The final extension was carried out at 72 °C for 3 min and the sample was cooled to 4 °C. (iv) Analyses of purified extension reaction products by MALDI-TOF–MS: The samples were then manually desalted by using 6 mg of clean resin and a dimple plate and subsequently transferred to a 384-well Spectro-CHIP (Sequenom, Inc.) using a nano dispenser. Mass spectrum was acquired by Compact Mass Spectrometer and analyzed by MassARRAYTyper 4.0 Software (Sequenom, Inc.). The PCR assay was arrayed with two no-template controls and four duplicated samples in each 384-well format as quality controls. All genotyping results were generated and checked by laboratory staff unaware of patient status.

### Statistical analyses

Values are expressed as mean ± standard deviation or as numbers and percentages. Differences in age between case group and control group were evaluated using the t-test. Differences in gender and frequencies of the alleles and genotypes between case group and control group were evaluated using the χ^2^-test. Mann–Whitney test was used to compare serum total IgE level between case and control groups, and between different genetic models in dominant or recessive models. Kruskal–Wallis test was used to compare serum total IgE level among different genotypes in co-dominant model.

Hardy–Weinberg Equilibrium (HWE) was tested by the Chi square test for goodness of fit with a Web program (http://ihg.gsf.de/cgi-bin/hw/hwa1.pl). It was tested in the control group by the Chi square test for goodness of fit, and a *P*-value of < 0.05 was considered to be statistically significant. Akaike’s information criteria were used to select the most parsimonious genetic model for each SNP [20]. Odds ratios (ORs) and 95% confidence intervals (CIs) were calculated by unconditional logistic regression analysis with adjustment for age and gender. The significance level was set at *P*-value of 0.05 after corrections with 100,000 permutations. All *P* values are two tailed. These analyses were conducted with Stata statistical package (version 10.0; Stata Corp LP, College Station, TX, USA).

## Results

### Demographic characteristics of study population

The demographic characteristics of the study population are shown in Table 1. Both the AR and control groups were well matched with respect to age and gender. The mean ages of the AR and control groups were 36 and 37 years old, respectively and both groups consisted of similar sex ratio (AR group = 51.6%/48.4% males/females; control group = 49.7%/50.3% males/females). There were no significant difference were found in both age (*P* = 0.167) or the ratios for males/females (*P* = 0.372) between the control and AR groups. The median total serum IgE measurements were significantly increased in AR group than control group (125.0 and 29.2 IU/ml respectively; *P* = 0.0000). Furthermore, 273 (35.8%), 168(22.0%), and 321 (42.2%) of AR subjects, respectively, were found to be allergic to HDMs alone, seasonal pollens alone, and mixed allergens.

**Table 1:** Demographic characteristics of study population

Demographic Index	Allergic rhinitis (n = 762)	Control (n = 760)
Age, Mean (Range) (years)	35.6 ± 12.7 (18–73)	37.4 ± 13.3 (18–78)
Sex, Male/Female, No. (%)	393 (51.6)/369 (48.4)	378 (49.7)/382 (50.3)
Duration(IQR) (months)	48.0(18.0–96.0)	–
Total IgE, Median (interquartile range) (kU/l)	125.0(61.95–277.0)	29.2(15.9–45.5)
Allergen category, no. (%)		
House dust mites	273 (35.8)	–
Seasonal pollens	168 (22.0)	–
Mixed allergens	321 (42.2)	–

### Individual SNP association analysis

The 58 SNP IDs, locations, and allele frequencies are also given in Additional file 1: Table S1. A total of 3 variants, including rs6586513, rs9266772, rs6906021 did not pass Hardy–Weinberg Equilibrium (HWE) test (*P *< 0.0001). The minor allele frequencies (MAF) of four selected SNPs, such as rs6673480, rs17513503, rs7032572 and rs1250761 could not satisfy association study (MAF < 0.01). The four SNPs, rs3860069, rs11680788, rs9303280 and rs12973620 failed in assay design. Thus, we finally enrolled 47 SNPs to process the next analysis (Additional file 1: Table S2).

The genotype distributions of 47 selected SNPs in the case group and control group are summarized in Table 2 and Additional file 1: Table S3. Based on the analysis of unadjusted codominant model, the allele frequencies of rs6554809 in DNAH5, rs1438673 in WDR36-CAMK4 loci and rs7775228 in HLA region were significantly different between the case group and control group: rs6554809: C>T (*P* = 0.006), rs1438673: T>C (*P* = 0.029), rs7775228: T>C (*P* = 0.001), and they remain associated after 100,000 permutations (P < 0.05). The distribution of the genotypes of rs6554809, rs1438673 and rs7775228 showed significant difference between cases and controls.

**Table 2:** Genotype frequencies of cases and controls and their associations with AR risk under co-dominant genetic model

Gene	SNP ID	Genotype	Case		Control		*P* (2 df)^a^	Logistic Regression		Armitage’s trend test
			No.	Frequency (%)	No.	Frequency (%)		OR (95% CI)	*P*^b^	
LPP	rs9865818	AA	325	42.71	361	47.50	0.052	1.000 (referent)		*0.018*
		GA	335	44.02	324	42.63		1.127 (0.903–1.407)	0.29	
		GG	101	13.27	75	9.87		*1.469 (1.041–2.074)*	*0.029*	
DNAH5	rs6554809	CC	541	71.18	593	78.03	*0.006*	1.000 (referent)		*0.007*
		TC	206	27.11	153	20.13		*1.597 (1.246–2.048)*	*0.000*	
		TT	13	1.71	14	1.84		1.235 (0.558–2.732)	0.603	
WDR36-CAMK4	rs1438673	TT	221	29.31	250	33.03	*0.029*	1.000 (referent)		*0.014*
		TC	382	50.66	393	51.92		1.045 (0.824–1.324)	0.718	
		CC	151	20.03	114	15.06		*1.396 (1.021–1.908)*	*0.037*	
HLA-DQB1-HLA-DQB2	rs7775228	TT	422	55.60	493	64.95	*0.001*	1.000 (referent)		*0.001*
		TC	275	36.23	216	28.46		*1.589 (1.263–1.999)*	*0.000*	
		CC	62	8.17	50	6.59		1.500 (0.996–2.259)	0.052	
CLEC16A	rs7203459	TT	633	83.07	604	79.58	0.177	1.000 (referent)		0.123
		TC	120	15.75	147	19.37		*0.731 (0.555–0.962)*	*0.025*	
		CC	9	1.18	8	1.05		0.962 (0.355–2.603)	0.939	

Italic values indicate significance of *P* value (*P* < 0.05)

^a^Global *P* values [2 degrees of freedom (df)]: genotype frequencies in AR and control group were compared using a χ^2^ test with 2 df

^b^*P* values from unconditional logistic regression analyses, adjusted for age and gender

Logistic regression analysis showed significantly related to AR risk compared with wild-type carriers in the codominant effect model assessed by AIC. The result showed that it was a risk factor with the SNP in rs9865818 in LPP (*P* = 0.029, OR = 1.469, 95% CI 1.041–2.074 for GG vs. AA); rs6554809 in DNAH5 (*P* = 0.000, OR = 1.597, 95% CI 1.246–2.048 for TC vs. CC); rs1438673 in WDR36-CAMK4 loci (*P* = 0.037, OR = 1.396, 95% CI 1.021–1.908 for CC vs. TT), rs7775228 in HLA region (*P* = 0.000, OR = 1.589, 95% CI 1.263–1.999 for TC vs. TT). However, it was a protective factor with the SNP in rs7203459 in CLEC16A (*P* = 0.025, OR = 0.731, 95% CI 0.555–0.962 for TC vs. TT). The detailed data was shown in Table 2. Moreover, 4 out of 5 polymorphisms’ trend analysis showed positive results (rs9865818 in LPP, rs6554809 in DNAH5, rs1438673 in WDR36-CAMK4 and rs7775228 in HLA).

Under dominant model, the significant AR risks were observed in rs6554809 in DNAH5 (*P* = 0.000, OR = 1.567, 95% CI 1.231–1.995) and in rs7775228 (*P* = 0.000, OR = 1.571, 95% CI 1.267–1.948), while protective effect was found in rs7203459 in CLEC16A (*P* = 0.030, OR = 0.743, 95% CI 0.568–0.971). In addition, under recessive model, rs7617456 in TMEM18 was also revealed with protective effect (*P* = 0.040, OR = 0.628, 95% CI 0.403–0.980), and rs1438673 was evaluated with AR risks (*P* = 0.029, OR = 1.359, 95% CI 1.031–1.790). The detailed data was shown in Table 3 and Additional file 1: Table S4.

**Table 3:** Association analysis of SNPs under dominant and recessive genetic model

Gene	SNP ID	Dominant model			Recessive model		
		Genetic model	OR (95% CI)	*P*^a^	Genetic model	OR (95% CI)	*P*^a^
TMEM108	rs7617456	(GA + AA) vs.GG	0.980 (0.794–1.209)	0.849	AA vs.(GG + GA)	*0.628 (0.403–0.980)*	*0.040*
DNAH5	rs6554809	(TC + TT) vs.CC	*1.567 (1.231–1.995)*	*0.000*	TT vs.(CC + TC)	1.098 (0.498-2.420)	0.817
WDR36-CAMK4	rs1438673	(TC + CC) vs.TT	1.124 (0.897–1.409)	0.308	CC vs.(TT + TC)	*1.359 (1.031–1.790)*	*0.029*
HLA-DQB1-HLA-DQB2	rs7775228	(TC + CC) vs.TT	*1.571 (1.267–1.948)*	*0.000*	CC vs.(TT + TC)	1.276 (0.855–1.906)	0.233
CLEC16A	rs7203459	(TC + CC) vs.TT	*0.743 (0.568–0.971)*	*0.030*	CC vs.(TT + TC)	1.017 (0.377–2.748)	0.973

Italic values indicate significance of *P* value (*P* < 0.05)

^a^*P* values from unconditional logistic regression analyses, adjusted for age and gender

The functions and effects of these positive SNPs were retrieved from HaploReg v4.1 tool (http://pubs.broadinstitute.org/mammals/haploreg/haploreg.php) and reviewed in Table 4. The frequencies of SNPs were different among the common ethnic groups based on 1000 Genomes. Consistent with the result of this study, these SNPs were associated with IgE grass sensitization [10], self-reported allergy [12], hay fever [21] and asthma [22]. The loci mentioned above can affect the expression of genes, which are located in the histone modified regulatory region.

**Table 4:** Functional annotation of positive SNPs in HaploReg v4.1 tool

SNP	Frequence in 1000 Genomes				Association phenotype with GWAS	eQTL	Histone markers
	AFR	AMR	ASN	EUR			
rs9865818	0.11	0.37	0.33	0.42	Allergic sensitization [11]	NA	H3K4me1_Enh H3K27ac_Enh
rs7617456	0.21	0.47	0.24	0.40	IgE grass sensitization [10]	NA	H3K4me3_Pro
rs6554809	0.49	0.25	0.12	0.16	IgE grass sensitization [10]	NA	H3K4me1_Enh
rs1438673	0.84	0.6	0.54	0.52	Asthma and hay fever [21] Self-reported allergy [12]	TSLP	H3K4me1_Enh
rs7775228	0.17	0.2	0.28	0.15	Asthma [22] IgE grass sensitization [10]	HLA-DPA1/HLA-DPA2/HLA-DQB1/HLA-DQB2	H3K4me1_Enh
rs7203459	0.18	0.21	0.09	0.26	Self-reported allergy [12]	LOC642755, C16orf75/SOCS1	H3K4me1_Enh H3K27ac_Enh

*AFR* African, *AMR* Amerindian, *ASN* Asian, *EUR* European, *eQTL* Expression quantitative trait loci, *NA* not available because of the lack of data

**Fig. 1:**
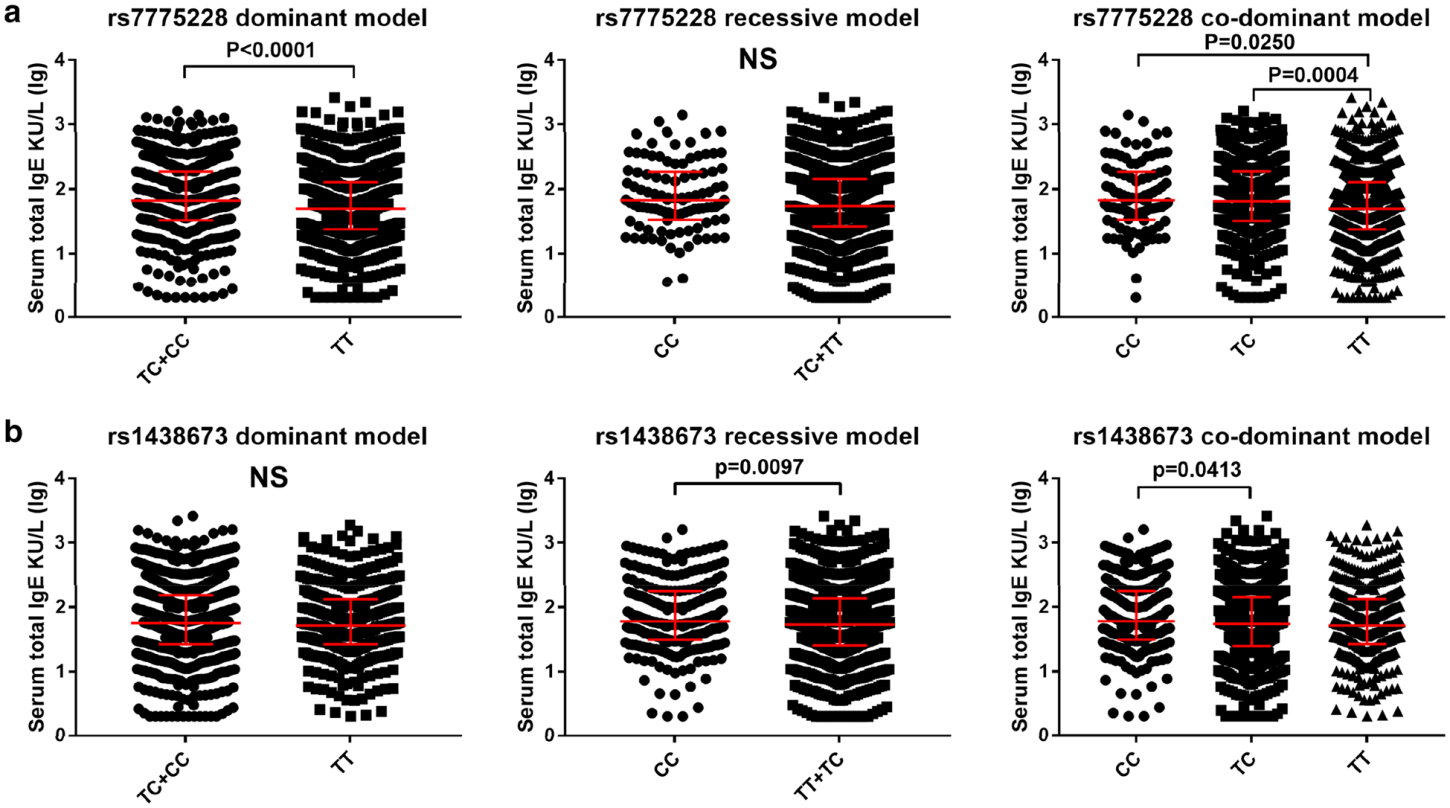
The association between genotypes and serum total IgE under recessive model, dominant model and co-dominant model analysis. **a** rs7775228 in HLA-DQB1-HLA-DQB2. **b** rs1438673 in WDR36-CAMK4. The level of serum IgE was described in median with interquartile range and Y axis was changed in logarithmic scale (lg = log_10_)

The above results suggested that allele “C” was regard as a risk allele in both rs7775228 and rs1438673 for AR subjects. Similarly with the result of AR risk, Under dominant model, the levels of serum total IgE significantly decreased in individuals with rs7775228_TT genotype compared to TC and CC carriers (median 49.8 KU/L vs. 66.7 KU/L, *P *< 0.0001). No significant association with serum total IgE was observed under recessive model for rs7775228. The co-dominant model analysis for rs7775228 found a significant decrease of serum total IgE among TT carries (median 49.8 KU/L) compared to CC (median 67.9 KU/L, *P *= 0.025) and TC carries (median 65.4 KU/L, *P *= 0.0004), respectively (Fig. 1a). Under recessive model analysis for rs1438673, the levels of serum total IgE significantly increased among CC carries (median 60.5 KU/L) compared to TC and TT carries (median 53.8 KU/L) (*P *= 0.0097), as well as under co-dominant model, significant elevation of serum total IgE was detected among CC carries (median 60.50 KU/L) comparing with TC carries (median 54.6 KU/L) (*P *= 0.0413) (Fig. 1b). No significant associations between another AR-associated SNPs and serum total IgE were noted under recessive, dominant and co-dominant genetic model (Additional file 1: Figure S3).

## Discussion

In present study we carried out a replication study of susceptibility variants associated with AR and allergy in AR as well as healthy population [11–13, 15]. We demonstrated that variants in or near LPP (rs9865818), DNAH5 (rs6554809), WDR36-CAMK4 (rs1438673), HLA (rs7775228) and CLEC16A (rs7203459) were significantly associated AR in Han Chinese.

Actually, it is almost 4 decades since an association between genetic variants in the HLA region and specific allergen sensitization was reported [23], and various loci in the HLA region have been implicated in candidate gene studies of asthma or allergic phenotypes [24]. A recent study investigated common genetic variant associations with prevalent AR and grass sensitization using existing GWAS data in 4 large European adult cohorts for AR (3933 self-reported cases vs. 8965 control subjects) and grass sensitization (2315 cases vs. 10,032 control subjects). Three loci reached genome-wide significance for either phenotype. The HLA variant rs7775228-C was strongly associated with grass sensitization and weakly with AR (*P*_grass_ = 1.6 × 10^−9^; *P*_AR_ = 8.0 × 10^−3^) and it cis-regulates HLA-DRB4^10^, similar with our present findings.

DNAH5 (rs6554809, *P *= 3.3 × 10^−6^) was firstly detected as a candidate gene for AR and grass sensitization through a genome-wide meta-analysis by Ramasamy et al. [10] in 2011, the same locus presented positive in present study. DNAH5 mutations are a common cause of primary ciliary dyskinesia with outer dynein arm defects [25] and DNAH5 has been regarded as a force generating of respiratory cilia. Sugier et al. combined GWAS and epitasis analysis and exhibited that DNAH5 and adhesion G protein-coupled receptor V1 (ADGRV1) interactions might represent a novel mechanism underlying for ciliary function in atopy [26].

Hinds et al. [12] conducted a meta-analysis of genome-wide associations with self-reported cat, dust-mite and pollen allergies in 53,862 individuals and identified 16 shared susceptibility loci with association *P *< 5×10^−8^, including rs9860547 in LPP (*P *= 1.2 × 10^−9^). Bønnelykke et al. [11] performed the first large-scale GWAS of allergic sensitization in 5789 affected individuals and 10,056 controls and followed up the top SNP at each of 26 loci in 6114 affected individuals and 9920 controls. They identified 10 susceptibility loci with significant association with allergic sensitization including rs9865818 in LPP (*P *= 3.4 × 10^−6^). Here we also replicated rs9865818 of LPP using diagnosed AR population. Several new allergy-associated loci are in or near genes involved in T-helper cell differentiation. The association between LPP and allergy may be mediated by an effect on the expression of BCL6 (B cell lymphoma 6), a transcription factor that represses the STAT6-mediated response to IL-4 and IL-13 and IgE class switching [27] and inhibits Th2 cell differentiation in a mouse model [28]. Conerning TSLP, a recent research suggested a role of TSLP in directly promoting T helper 2(Th2) cell effector function and support the notion of TSLP as a key driver of Th2 inflammation [29], while the crucial effects of Th2 lineage in the pathogenesis of allergy have been always highlighted with the associations identified in or near key Th2 pathways genes. Interestingly, here we couldn’t successfully repeat the allergy susceptible loci in TSLP while we identified a signal (rs1438673) in 5q22.1 in WDR36-CAMK4 intergenic region, which are about 50,000 bp far away from TSLP gene. The Genotype-Tissue Expression (GTEx) data exhibit a strong association between rs1438673 genotype and TSLP expression. Likewise, another our previous study also demonstrated that the SNPs in TSLP locus ensured complete genetic coverage were not associated with AR susceptibility in Chinese subjects [30].

In this study, we found that the SNPs in rs1438673 and in rs7775228 had strongly association with serum total IgE in AR group. The variant near the HLA-DQB1 was regarded as a predictor of total serum IgE levels in multiple race-ethnic groups, which was identified by an independently meta-analysis [31]. The serum total IgE level was used to diagnosis of AR in the in vitro, and high total IgE level suggests that in vitro testing would confirm specific sensitizations in AR patients [32]. The serum total IgE might play a role as a mediator in the development of allergic diseases. The high serum total IgE concentration was associated with the risk for allergic sensitization [33], In addition, high serum total IgE increased macrophage expression of TLR4 in induced sputum in asthmatic subjects [34]. This outcome may result from a link between innate immunity and IgE-mediated adaptive immune responses in asthma. The variation of serum total IgE also showed have an association with asthma control [35], which might also increase the risk of AR. Besides, our findings also reinforced the notion that there was a shared genetic etiology of allergic and autoimmune disease, with discovered susceptibility loci for allergy, many of which were previously associated with autoimmune disease [12]. In present study, we exhibited rs7203459 in CLEC16A which had been proven to be associated with type 1 diabetes mellitus [36–38], multiple sclerosis [39] and other autoimmune disease [40] was associated with AR (OR = 0.731), indicating such risk allele for autoimmune disease seemed to be protective against allergy in Han Chinese. Comparably, previous study reported that the data in same locus in CLEC16A revealed that autoimmune disease and allergy were associated with the same risk alleles (OR = 1.07) [12]. Such inconsistent results to some degree suggested that the complex context of autoimmune diseases as well as allergy as many autoimmune diseases were associated with increased activation of Th1 responses, whereas allergy had been associated with Th2 activity.

It is striking that in the present study, variants in only 5 of 58 previously identified candidate genes demonstrated significant association with Han Chinese AR. Of these variants, rs6554809-T allele in DNAH5 was the risk factor for AR in our study, which was opposite to Ramasamy et al. [10]. These observations suggest that the genetic determination of AR as well as allergic diseases might perhaps be more complicated than what we suspected. Although AR and other allergic diseases commonly coexist and share some susceptibility genes, there appeared to be more genetic heterogeneity among patients with atopic disorders [41]. Most GWASs enrolled admixture of subjects with diverse phenotypes and it is difficult to determine disease-specific or overlapping genes with confidence. In the present study, under the premise of a limited study population, we specifically excluded subjects with comorbid asthma, eczema and other allergic disease to reduce the effect of comorbidity as potential confounding factors and mainly focused on these selected genes involved in susceptibility to pure AR. It’s worthy to mention that all the AR individuals here were diagnosed AR in terms of skin test response and serum allergen specific IgE, guaranteeing the clear diagnosis of our study population. However, it is to be noted that our study showed a poor reproducibility of reported associations from 6 previous genome-wide studies in AR and allergy, and especially lack of association for 5 SNPs previously associated with asthma. In addition to the “allergic disease genes”, there are “tissue-specific genes” that contribute to the expression to a certain atopic disease [41], such as GSDMB locus, where none of association was detected in the present study, exerting greatest effect in bronchi tissues. Possibly different combinations of susceptibility genes, such as gene–gene interactions, are involved in different allergic phenotypes [1]. In addition, the nature of variant susceptibility in different populations exhibits heterogeneity. On the one hand, the levels of linkage disequilibrium exist different among distinct populations, such as the risk allele of rs6554809 in DNAH5 in Chinese population was lower than European population (0.09 vs. 0.16). On the other hand, investigation from recent association of rare and low-frequency variants with asthma suggested some loci are ethnicity specific, including associated variants in GRASP and GSDMB in patients with Latino ancestry and variants in MTHFR in patients with African ancestry [42]. Convincingly replication in different populations would be necessary to identify some loci or genes that are more frequently involved in the pathogenesis of AR and allergy and would provide some potential targeting of preventative treatments and are therefore of great interest in the clinical perspective. For example, we reported the rs777528 (CC + CT) genotypes in HLA-DQB1-HLA-DQB2 loci presented risk role for AR and were associated with an increased total serum IgE levels, suggesting central role in allergic and immune response.

However, the findings of our study are slightly limited, particularly in view of associations in only 6 genome-wide studies due to a limited number of SNPs in two genotyping assays. Furthermore, this study is somewhat limited in the sample size and the results did not exclude the potential false positive and negative associations, although we enrolled well-diagnosed AR to avoid population stratification as well as adjustment for multiple testing involved in 47 SNPs. Further studies would also be performed in larger cohorts and assess associations in a larger number of loci for identification of susceptibility to AR.

Intriguingly, whereas, it’s indubitable that the analysis driven by statistical and knowledge-based evidence represents a promising approach for identifying new genes or targets involved in complex traits and an important next step will be to more carefully characterize the extent to which indi-vidual associations lead to a global predisposition to allergy compared to effects on specific target tissues, such as skin, lung or mucosa.

## Conclusions

In summary, here we replicated shared and AR-specific susceptibility loci in LPP, DNAH5, HLA, CLEC16A and WDR36-CAMK4 which were also plausible candidates in terms of their biological function in the development of AR. Further understanding the molecular mechanisms underlying these associations may provide new insights into the etiology of allergic disease in Han Chinese.

## Supplementary information

**Additional file 1.** Additional tables and figures.

## Data Availability

The datasets used and/or analyzed during the current study are available from the corresponding author on reasonable request.

## Abbreviations

AR: Allergic rhinitis
ARIA: Allergic Rhinitis and its Impact on Asthma
CIs: Confidence intervals
Der f: Dermatophagoides farinae
Der p: Dermatophagoides pteronyssinus
GINA: Global Initiative for Asthma
GWASs: Genome-wide association studies
HDM: House dust mite
HWE: Hardy–Weinberg Equilibrium
IgE: Immunoglobulin E
ORs: Odds ratios
sIgE: Specific IgE
SNPs: Single nucleotide polymorphisms
