# Supplementary material for: Replication study of susceptibility variants associated with allergic rhinitis and allergy in Han Chinese
Source: Allergy Asthma Clin Immunol. 2020 Feb 11;16:13. doi: 10.1186/s13223-020-0411-9 (PMC7014941; doi:10.1186/s13223-020-0411-9)

**Table S1. SNPs associated with AR and allergy phenotypes in 6 genome-wide studies.**

| Reference | Gene | SNP_ID | Chr.Position ^a^  (GRCh38.p12) | Genic location | Minor/major allele | Population | Associated phenotype | Reported  *P* value | Reported OR (95% CI) |
| --- | --- | --- | --- | --- | --- | --- | --- | --- | --- |
| Ramasamy, *et al.* (2011) | TSLP | rs1898671 | chr5:111072304 | Intron | T/C | European-the British 1958 Birth Cohort; the follow-up of the European Community Respiratory Health Survey; the Northern Finland Birth Cohort of 1966; and the Swiss Study on Air Pollution and Lung Disease in Adults | AR | 5.2×10^-6^ | 1.15 (1.08-1.22) |
|  | PPM1A-DHRS7 | rs216518 | chr14:60216712 | Intergenic region | A/C |  |  | 1.9×10^-6^ | 1.21 (1.12-1.31) |
|  | GLI3 | rs4724100 | chr7:42225080 | Intron | C/T |  |  | 2.2×10^-6^ | 1.14 (1.08-1.21) |
|  | ENTPD6 | rs1044573 | chr20:25226018 | 3’ UTR | G/A |  |  | 9.7×10^-7^ | 1.15 (1.09-1.21) |
|  | SEMA6A-TNFAIP8 | rs6898653 | chr5:116639960 | Intergenic region | G/A |  |  | 1.0×10^-6^ | 1.23 (1.13-1.34) |
|  | MST1L-CROCC | rs6586513 | chr1:16889836 | Intergenic region | A/C |  |  | 3.9×10^-6^ | 1.24 (1.13-1.36) |
|  | CLEC16A | rs887864 | chr16:11065028 | Intron | G/A |  |  | 1.1×10^-6^ | 1.16 (1.09-1.23) |
|  | TMEM108 | rs7617456 | chr3:133009059 | Intergenic region | A/G |  | Grass sensitization | 3.3×10^-6^ | 1.18 (1.10-1.26) |
|  | NOD1 | rs7789045 | chr7:30454406 | 5' near gene | A/T |  |  | 6.2×10^-5^ | 1.15 (1.08-1.24) |
|  | IL2 | rs2069772 | chr4:122451978 | Intron | C/T |  |  | 1.1×10^-6^ | 1.19 (1.11-1.28) |
|  | HLA-DQB1-HLA-DQB2 | rs7775228 | chr6:32690302 | Intergenic region | C/T |  |  | 1.6×10^-9^ | 1.33 (1.21-1.45) |
|  | C16orf72-GRIN2A | rs631208 | chr16:9305867 | Intergenic region | G/A |  |  | 2.0×10^-6^ | 1.18 (1.10-1.26) |
|  | TLR1-TLR6 | rs3860069 | chr4:38860500 | Intergenic region | C/A |  |  | 4.4×10^-5^ | 1.21 (1.11-1.33) |
|  | EPS15 | rs6673480 | chr1:51393570 | Intron | T/C |  |  | 2.2×10^-6^ | 1.35 (1.19-1.52) |
|  | DNAH5 | rs6554809 | chr5:13740867 | Intron | T/C |  |  | 3.3×10^-6^ | 1.29 (1.16-1.43) |
|  | ABL2 | rs1325195 | chr1:179102621 | 3’ UTR | G/A |  |  | 4.6×10^-6^ | 1.17 (1.10-1.26) |
|  | C11orf30-LRRC32 | rs2155219 | chr11:76588150 | Intergenic region | T/G |  | AR and Grass sensitization | 3.8×10^−8^forAR and9.4×10^-9^ for grass sensitization | 1.17 (1.11-1.24) for AR and 1.22 (1.14-1.31)for grass sensitization |
|  | SLC25A46-TSLP | rs17513503 | chr5:110810746 | Intergenic region | G/C |  |  | 7.4×10^-7^ forAR and 1.2×10^-8^ for grass sensitization | 1.28 (1.16-1.41) for AR and 1.39 (1.24-1.56) for grass sensitization |
| Bønnelykke, *et al.* (2013) | C11orf30-LRRC32 | rs2155219 | chr11:76588150 | Intergenic region | T/G | European | Allergen sensitization | 1.4×10^−18^ | 1.18(1.13-1.22) |
|  | STAT6 | rs1059513 | chr12:57095926 | 3’ UTR | C/T |  |  | 1.0×10-^14^ | 1.30(1.21-1.39) |
|  | SLC25A46-TSLP | rs10056340 | chr5:110854353 | Intergenic region | G/T |  |  | 5.2×10^-14^ | 0.83(0.78-0.87) |
|  | HLA-DQB1-AS1 | rs6906021 | chr6:32658534 | 2KB Upstream | C/T |  |  | 2.2×10^−12^ | 0.87(0.83-0.90) |
|  | IL1RL1 | rs3771175 | chr2:102343750 | 3’ UTR | A/G |  |  | 4.9×10^-11^ | 0.83(0.78-0.88) |
|  | TLR1- TLR6 | rs17616434 | chr4:38811255 | Intergenic region | C/T |  |  | 5.2×10^-11^ | 1.23(1.18-1.29) |
|  | LPP | rs9865818 | chr3:188354725 | Intron | G/A |  |  | 2.7×10^-10^ | 0.89(0.86-0.92) |
|  | PVT1 | rs4410871 | chr8:127802783 | Intron | T/C |  |  | 5.4×10^-10^ | 1.14(1.09-1.19) |
|  | ADAD1-IL2 | rs17454584 | chr4:122432277 | Intergenic region | G/A |  |  | 5.5×10^-10^ | 0.87(0.83-0.91) |
|  | HLA-B-MICA | rs6932730 | chr6:31386405 | Intergenic region | C/T |  |  | 4.2×10^-8^ | 1.14(1.09-1.20) |
| Hinds, *et al.* (2013) | TLR1- TLR6 | rs2101521 | chr4:38809930 | Intergenic region | G/A | Individuals having >97% European ancestry and pregnant women resident in Avon, UK | Self-reportedallergic sensitization- for pollen allergy, dust mite  Allergy and cat allergy | 5.3×10^-21^ | 1.15(1.11-1.18) |
|  | WDR36-CAMK4 | rs1438673 | chr5:111131801 | Intergenic region | C/T |  |  | 2.3×10^−20^ | 1.12(1.09-1.14) |
|  | C11orf30-LRRC32 | rs2155219 | chr11:76588150 | Intergenic region | G/T |  |  | 1.6×10^−19^ | 1.11(1.09-1.14) |
|  | IL1RL1-IL1RL2 | rs10189629 | chr2:102263004 | Intergenic region | A/C |  |  | 1.8×10^−16^ | 1.16(1.12-1.20) |
|  | HLA-DQB1-AS1 | rs6906021 | chr6:32658534 | 2KB Upstream | C/T |  |  | 7.1×10^−15^ | 1.10(1.07-1.13) |
|  | HLA-B-MICA | rs9266772 | chr6:31384336 | Intergenic region | C/T |  |  | 3.2×10^−12^ | 1.11(1.08-1.14) |
|  | PTGER4-DAB2 | rs7720838 | chr5:40486794 | Intergenic region | G/T |  |  | 8.2×10^−11^ | 1.08(1.06-1.11) |
|  | PLCL1 | rs10497813 | chr2:198049348 | Intron | G/T |  |  | 6.1×10^−10^ | 1.08(1.05-1.10) |
|  | LPP | rs9860547 | chr3:188411191 | Intron | A/G |  |  | 1.2×10^−9^ | 1.08(1.05-1.10) |
|  | GSDMB | rs9303280 | chr17:39917778 | Intron | T/C |  |  | 8.9×10^-9^ | 1.07(1.05-1.10) |
|  | RANBP6-IL33 | rs7032572 | chr9:6172380 | Intergenic region | G/A |  |  | 1.7×10^−9^ | 1.12(1.08-1.16) |
|  | NFATC2 | rs6021270 | chr20:51524725 | Intron | C/T |  |  | 6.9×10^−9^ | 1.16(1.11-1.23) |
|  | SMAD3 | rs17228058 | chr15:67157967 | Intron | G/A |  |  | 1.2×10^−8^ | 1.08(1.05-1.11) |
|  | GATA3-CELF2 | rs962993 | chr10:9011169 | Intergenic region | T/C |  |  | 1.5×10^−8^ | 1.07(1.05-1.10) |
|  | ADAD1 | rs17388568 | chr4:122408207 | Intron | A/G |  |  | 3.9×10^−8^ | 1.07(1.05-1.10) |
|  | TTC6 | rs1998359 | chr14:37607943 | Intron | G/C |  |  | 4.8×10^−8^ | 1.08(1.05-1.12) |
|  | TPD52-ZBTB10 | rs6473223 | chr8:80355920 | Intergenic region | T/C |  |  | 7.7×10^−8^ | 1.07(1.04-1.10) |
|  | RNF144A-ID2 | rs10174949 | chr2:8302118 | Intergenic region | A/G |  |  | 1.0×10^−7^ | 1.07(1.05-1.10) |
|  | CLEC16A | rs7203459 | chr16:11136846 | Intron | C/T |  |  | 2.0×10^−7^ | 1.07(1.04-1.10) |
|  | IL21R-IL4R | rs2107357 | chr16:27399508 | Intergenic region | A/G |  |  | 3.3×10^−7^ | 1.09(1.06-1.13) |
|  | PEX14 | rs2056417 | chr1:10521601 | Intron | A/G |  |  | 3.7×10^−7^ | 1.07(1.04-1.10) |
|  | KIRREL3-ETS1 | rs10893845 | chr11:128316987 | Intergenic region | G/T |  |  | 6.4×10^−7^ | 1.09(1.04-1.09) |
| Bunyavanich, *et al.* (2014) | TWIST1-TMEM196 | rs7780001 | chr7:19438358 | Intergenic region | A/T | European American, Latino and African | AR | 2.0×10^-8^ | 0.73(0.62-0.84) |
|  | LINC00486 | rs11680788 | chr2:32834029 | Intron | T/C |  |  | 3.8×10^−8^ | 0.47(0.19-0.82) |
|  | DLG1-BDH1 | rs6583203 | chr3:197352715 | Intergenic region | T/C |  |  | 1.4×10^−8^ | 1.65(1.48-1.83) |
|  | EIF5AL1-ZCCHC24 | rs1250761 | chr10:79500204 | Intergenic region | A/G |  |  | 4.5×10^-9^ | 1.80（1.61-2.00） |
|  | ZNF776 | rs12973620 | chr19:57738275 | Intergenic region | C/G |  |  | 5.0×10^-8^ | 1.56(1.40-1.72) |
| Bønnelykke, *et al.* (2014) | GSDMB | rs2305480 | chr17:39905943 | Missense Variant | A/G | European | Early childhood asthma with severe exacerbations | 6.4×10^−23^ | 1.32(1.23-1.39) |
|  | IL33 | rs928413 | chr9:6213387 | 2KB Upstream | A/G |  |  | 8.8×10^−13^ | 1.24(1.17-1.32) |
|  | RAD50 | rs6871536 | chr5:132634182 | Intron | C/T |  |  | 7.6×10^−7^ | 1.17(1.10-1.25) |
|  | IL1R1 | rs1558641 | chr2:102149405 | Intron | A/G |  |  | 6.9×10^−9^ | 1.11(1.04-1.19) |
|  | CDHR3 | rs6967330 | chr7:106018005 | Missense Variant | A/G |  |  | 2.7×10^−14^ | 1.26(1.18-1.33) |
| Dizier, *et al.* (2014) | C1orf87-NFIA | rs12122228 | chr1:60890848 | Intergenic region | T/G | European | Asthma with AR | 2.0×10^-6^ | 0.61(0.47-0.80) |

a. SNP position in the NCBI dbSNP database (http://www.ncbi.nlm.nih.gov/SNP).

**Table S2. Allele frequencies and HWE testing for replicated SNPs.**

| Gene | SNP_ID | Minor/ major  Allele | MAF ^a^ | | | *P*^c^ | *P* for HWE^d^ | Genotyping Rate (%)^e^ | Analysis |
| --- | --- | --- | --- | --- | --- | --- | --- | --- | --- |
|  |  |  | NCBI ^b^ | AR | Control |  |  |  |  |
| PEX14 | rs2056417 | A/G | 0.140 | 0.188 | 0.189 | 0.898 | 0.112 | 100.00 | 47 SNPs were analyzed in this study |
| C1orf87-NFIA | rs12122228 | T/G | 0.342 | 0.237 | 0.232 | 0.763 | 0.685 | 100.00 |  |
| ABL2 | rs1325195 | G/A | 0.442 | 0.501 | 0.493 | 0.690 | 0.888 | 100.00 |  |
| RNF144A-ID2 | rs10174949 | A/G | 0.275 | 0.237 | 0.263 | 0.094 | 0.413 | 100.00 |  |
| IL1R1 | rs1558641 | T/C | 0.275 | 0.233 | 0.223 | 0.519 | 0.834 | 98.36 |  |
| IL1RL1-IL1RL2 | rs10189629 | A/C | 0.092 | 0.082 | 0.086 | 0.727 | 0.469 | 100.00 |  |
| IL1RL1 | rs3771175 | A/T | 0.117 | 0.081 | 0.091 | 0.360 | 0.319 | 99.93 |  |
| PLCL1 | rs10497813 | T/G | 0.292 | 0.230 | 0.242 | 0.438 | 0.835 | 98.69 |  |
| TMEM108 | rs7617456 | A/G | 0.225 | 0.236 | 0.257 | 0.171 | 0.133 | 99.80 |  |
| LPP | rs9865818 | G/A | 0.367 | 0.353 | 0.312 | 0.016 | 0.853 | 99.93 |  |
|  | rs9860547 | A/G | 0.450 | 0.406 | 0.373 | 0.062 | 0.969 | 99.93 |  |
| DLG1-BDH1 | rs6583203 | T/C | 0.033 | 0.033 | 0.036 | 0.610 | 0.300 | 100.00 |  |
| TLR1- TLR6 | rs2101521 | G/A | 0.333 | 0.377 | 0.361 | 0.377 | 0.544 | 100.00 |  |
|  | rs17616434 | T/C | 0.317 | 0.369 | 0.352 | 0.335 | 0.650 | 100.00 |  |
| ADAD1 | rs17388568 | A/G | 0.100 | 0.142 | 0.130 | 0.322 | 0.472 | 99.93 |  |
| ADAD1-IL2 | rs17454584 | G/A | 0.075 | 0.142 | 0.130 | 0.330 | 0.320 | 100.00 |  |
| IL2 | rs2069772 | C/T | 0.100 | 0.144 | 0.129 | 0.236 | 0.445 | 100.00 |  |
| DNAH5 | rs6554809 | T/C | 0.092 | 0.153 | 0.119 | 0.007 | 0.265 | 99.87 |  |
| PTGER4-DAB2 | rs7720838 | T/G | 0.158 | 0.247 | 0.270 | 0.159 | 0.543 | 100.00 |  |
| SLC25A46-TSLP | rs10056340 | G/T | 0.142 | 0.152 | 0.138 | 0.248 | 0.181 | 100.00 |  |
| TSLP | rs1898671 | T/C | 0.050 | 0.046 | 0.045 | 0.943 | 0.716 | 100.00 |  |
| WDR36-CAMK4 | rs1438673 | C/T | 0.458 | 0.454 | 0.410 | 0.016 | 0.044 | 99.28 |  |
| SEMA6A-TNFAIP8 | rs6898653 | G/A | 0.267 | 0.143 | 0.155 | 0.352 | 0.851 | 99.93 |  |
| RAD50 | rs6871536 | C/T | 0.208 | 0.186 | 0.168 | 0.190 | 0.739 | 99.87 |  |
| HLA-B-MICA | rs6932730 | C/T | 0.150 | 0.144 | 0.144 | 0.982 | 0.879 | 98.95 |  |
| TWIST1-TMEM196 | rs7780001 | T/A | 0.292 | 0.238 | 0.220 | 0.250 | 0.153 | 99.93 |  |
| NOD1 | rs7789045 | T/A | 0.333 | 0.380 | 0.378 | 0.874 | 0.183 | 99.93 |  |
| GLI3 | rs4724100 | T/C | 0.325 | 0.243 | 0.238 | 0.765 | 0.327 | 100.00 |  |
| CDHR3 | rs6967330 | A/G | 0.075 | 0.074 | 0.062 | 0.178 | 0.953 | 100.00 |  |
| TPD52-ZBTB10 | rs6473223 | C/T | 0.433 | 0.393 | 0.391 | 0.911 | 0.046 | 99.87 |  |
| PVT1 | rs4410871 | T/C | 0.358 | 0.353 | 0.351 | 0.900 | 0.610 | 99.93 |  |
| IL33 | rs928413 | G/A | 0.050 | 0.065 | 0.080 | 0.119 | 0.557 | 100.00 |  |
| GATA3-CELF2 | rs962993 | T/C | 0.175 | 0.091 | 0.078 | 0.178 | 0.768 | 100.00 |  |
| C11orf30-LRRC32 | rs2155219 | G/T | 0.483 | 0.431 | 0.444 | 0.468 | 0.247 | 99.93 |  |
| KIRREL3-ETS1 | rs10893845 | G/T | 0.300 | 0.242 | 0.251 | 0.583 | 0.788 | 98.16 |  |
| STAT6 | rs1059513 | C/T | 0.092 | 0.068 | 0.075 | 0.462 | 0.909 | 99.67 |  |
| TTC6 | rs1998359 | G/C | 0.092 | 0.108 | 0.109 | 0.933 | 0.443 | 100.00 |  |
| SMAD3 | rs17228058 | G/A | 0.217 | 0.019 | 0.022 | 0.518 | 0.527 | 99.87 |  |
| C16orf72-GRIN2A | rs631208 | G/A | 0.208 | 0.265 | 0.272 | 0.651 | 0.507 | 100.00 |  |
| CLEC16A | rs887864 | G/A | 0.175 | 0.196 | 0.213 | 0.265 | 0.561 | 100.00 |  |
|  | rs7203459 | C/T | 0.117 | 0.091 | 0.107 | 0.120 | 0.776 | 99.93 |  |
| IL21R-IL4R | rs2107357 | A/G | 0.225 | 0.285 | 0.274 | 0.503 | 0.103 | 99.80 |  |
| GSDMB | rs2305480 | A/G | 0.350 | 0.283 | 0.302 | 0.248 | 0.737 | 98.95 |  |
| ENTPD6 | rs1044573 | A/G | 0.075 | 0.061 | 0.072 | 0.236 | 0.254 | 100.00 |  |
| NFATC2 | rs6021270 | C/T | 0.050 | 0.060 | 0.052 | 0.315 | 0.968 | 100.00 |  |
| HLA-DQB1-HLA-DQB2 | rs7775228 | C/T | 0.308 | 0.263 | 0.208 | 0.000 | 0.0002 | 99.74 |  |
| PPM1A-DHRS7 | rs216518 | A/C | 0.067 | 0.037 | 0.041 | 0.563 | 0.0005 | 100.00 |  |
| HLA-B-MICA | rs9266772 | C/T | 0.150 | 0.109 | 0.120 | 0.321 | < 0.0001 | 100.00 | HWE test *P*<0.0001 |
| HLA-DQB1-AS1 | rs6906021 | C/T | 0.442 | 0.443 | 0.405 | 0.035 | < 0.0001 | 96.91 |  |
| MST1L-CROCC | rs6586513 | A/C | 0.178 | 0.160 | 0.145 | 0.261 | < 0.001 | 99.47 |  |
| EIF5AL1-ZCCHC24 | rs1250761 | A/G | 0.000 | 0.000 | 0.000 | **-** | **-** | **-** | MAF<0.01 |
| RANBP6-IL33 | rs7032572 | G/A | 0.183 | 0.004 | 0.004 | 0.996 | 0.913 | 100.00 |  |
| SLC25A46-TSLP | rs17513503 | G/C | 0.022 | 0.000 | 0.001 | 0.856 | 0.985 | 99.61 |  |
| EPS15 | rs6673480 | T/C | 0.000 | 0.001 | 0.003 | 0.455 | 0.942 | 100.00 | the genotyping assays failed for these SNPs |
| TLR1-TLR6 | rs3860069 | NA | NA | NA | NA | NA | NA | NA |  |
| LINC00486 | rs11680788 | NA | NA | NA | NA | NA | NA | NA |  |
| GSDMB | rs9303280 | NA | NA | NA | NA | NA | NA | NA |  |
| ZNF776 | rs12973620 | NA | NA | NA | NA | NA | NA | NA |  |

a. MAF, minor allele frequency.

b. MAF for Chinese in the NCBI dbSNPs database.

c. *P* value for difference in allele frequencies between AR and control group.

d. HWE, Hardy–Weinberg equilibrium in control group.

e. Genotyping Rate (%) is the percentage of successfully genotyped individuals in the current study.

f. NA, not available because of the failed to replicate in this study.

**Table S3.** Genotype frequencies of 47 among cases and controls and their associations with AR risk under co-dominant genetic model.

| Gene | SNP ID | Genotype | Case | | Control | | *P* (2 df)^a^ | logistic Regression | | Armitage's trend test |
| --- | --- | --- | --- | --- | --- | --- | --- | --- | --- | --- |
|  |  |  | No. | Frequency | No. | Frequency |  | OR (95%CI) | *P*^b^ |  |
| PEX14 | rs2056417 | GG | 506 | 66.40% | 506 | 66.58% | 0.849 | 1.000 (referent) |  | 0.901 |
|  |  | AG | 226 | 29.66% | 220 | 28.95% |  | 0.996 (0.791-1.254) | 0.973 |  |
|  |  | AA | 30 | 3.94% | 34 | 4.47% |  | 0.881 (0.522-1.487) | 0.636 |  |
| C1orf87-NFIA | rs12122228 | GG | 442 | 58.01% | 446 | 58.68% | 0.954 | 1.000 (referent) |  | 0.761 |
|  |  | GT | 279 | 36.61% | 275 | 36.18% |  | 1.027 (0.825-1.279) | 0.811 |  |
|  |  | TT | 41 | 5.38% | 39 | 5.13% |  | 1.049 (0.652-1.686) | 0.844 |  |
| ABL2 | rs1325195 | GG | 198 | 25.98% | 196 | 25.79% | 0.714 | 1.000 (referent) |  | 0.693 |
|  |  | GA | 365 | 47.90% | 378 | 49.74% |  | 0.954 (0.741-1.228) | 0.715 |  |
|  |  | AA | 199 | 26.12% | 186 | 24.47% |  | 1.094 (0.818-1.462) | 0.547 |  |
| RNF144A-ID2 | rs10174949 | GG | 450 | 59.06% | 417 | 54.87% | 0.244 | 1.000 (referent) |  | 0.100 |
|  |  | GA | 263 | 34.51% | 286 | 37.63% |  | 0.867 (0.695-1.082) | 0.208 |  |
|  |  | AA | 49 | 6.43% | 57 | 7.50% |  | 0.817 (0.538-1.241) | 0.343 |  |
| IL1R1 | rs1558641 | CC | 431 | 57.31% | 449 | 60.27% | 0.229 | 1.000 (referent) |  | 0.508 |
|  |  | CT | 292 | 38.83% | 260 | 34.90% |  | 1.184 (0.949-1.476) | 0.134 |  |
|  |  | TT | 29 | 3.86% | 36 | 4.83% |  | 0.784 (0.465-1.322) | 0.361 |  |
| IL1RL1-IL1RL2 | rs10189629 | CC | 645 | 84.65% | 634 | 83.42% | 0.34 | 1.000 (referent) |  | 0.729 |
|  |  | CA | 109 | 14.30% | 122 | 16.05% |  | 0.839 (0.628-1.121) | 0.236 |  |
|  |  | AA | 8 | 1.05% | 4 | 0.53% |  | 1.446 (0.423-4.936) | 0.556 |  |
| IL1RL1 | rs3771175 | TT | 643 | 84.49% | 626 | 82.37% | 0.374 | 1.000 (referent) |  | 0.357 |
|  |  | AT | 112 | 14.72% | 130 | 17.11% |  | 0.817 (0.615-1.086) | 0.164 |  |
|  |  | AA | 6 | 0.79% | 4 | 0.53% |  | 1.077 (0.296-3.921) | 0.91 |  |
| PLCL1 | rs10497813 | GG | 454 | 60.53% | 431 | 57.31% | 0.245 | 1.000 (referent) |  | 0.445 |
|  |  | GT | 247 | 32.93% | 278 | 36.97% |  | 0.825 (0.659-1.032) | 0.092 |  |
|  |  | TT | 49 | 6.53% | 43 | 5.72% |  | 1.057 (0.678-1.648) | 0.806 |  |
| TMEM108 | rs7617456 | GG | 438 | 57.63% | 427 | 56.26% | 0.063 | 1.000 (referent) |  | 0.173 |
|  |  | GA | 286 | 37.63% | 274 | 36.10% |  | 1.051 (0.843-1.310) | 0.658 |  |
|  |  | AA | 36 | 4.74% | 58 | 7.64% |  | 0.640 (0.407-1.007) | 0.053 |  |
| LPP | rs9865818 | AA | 325 | 42.71% | 361 | 47.50% | 0.052 | 1.000 (referent) |  | **0.018** |
|  |  | GA | 335 | 44.02% | 324 | 42.63% |  | 1.127 (0.903-1.407) | 0.29 |  |
|  |  | GG | 101 | 13.27% | 75 | 9.87% |  | **1.469 (1.041-2.074)** | **0.029** |  |
| LPP | rs9860547 | GG | 274 | 36.01% | 299 | 39.34% | 0.155 | 1.000 (referent) |  | 0.064 |
|  |  | GA | 356 | 46.78% | 355 | 46.71% |  | 1.080 (0.860-1.357) | 0.507 |  |
|  |  | AA | 131 | 17.21% | 106 | 13.95% |  | 1.330 (0.971-1.823) | 0.076 |  |
| DLG1-BDH1 | rs6583203 | TT | 712 | 93.44% | 705 | 92.76% | 0.603 | 1.000 (referent) |  | 0.603 |
|  |  | CT | 50 | 6.56% | 55 | 7.24% |  | 0.845 (0.560-1.276) | 0.424 |  |
|  |  | CC | 0 | 0.00% | 0 | 0.00% |  | NA^c^ | NA^c^ |  |
| TLR1-TLR6 | rs2101521 | AA | 299 | 39.24% | 314 | 41.32% | 0.677 | 1.000 (referent) |  | 0.381 |
|  |  | AG | 352 | 46.19% | 343 | 45.13% |  | 1.079 (0.862-1.351) | 0.507 |  |
|  |  | GG | 111 | 14.57% | 103 | 13.55% |  | 1.165 (0.844-1.608) | 0.354 |  |
| TLR1-TLR6 | rs17616434 | CC | 306 | 40.16% | 322 | 42.37% | 0.631 | 1.000 (referent) |  | 0.338 |
|  |  | TC | 350 | 45.93% | 341 | 44.87% |  | 1.077 (0.861-1.347) | 0.517 |  |
|  |  | TT | 106 | 13.91% | 97 | 12.76% |  | 1.174 (0.846-1.630) | 0.336 |  |
| ADAD1 | rs17388568 | GG | 561 | 73.72% | 578 | 76.05% | 0.574 | 1.000 (referent) |  | 0.326 |
|  |  | GA | 184 | 24.18% | 167 | 21.97% |  | 1.138 (0.888-1.457) | 0.307 |  |
|  |  | AA | 16 | 2.10% | 15 | 1.97% |  | 1.079 (0.511-2.279) | 0.841 |  |
| ADAD1-IL2 | rs17454584 | AA | 562 | 73.75% | 578 | 76.05% | 0.583 | 1.000 (referent) |  | 0.336 |
|  |  | GA | 183 | 24.02% | 166 | 21.84% |  | 1.135 (0.886-1.455) | 0.316 |  |
|  |  | GG | 17 | 2.23% | 16 | 2.11% |  | 1.092 (0.529-2.254) | 0.811 |  |
| IL2 | rs2069772 | TT | 560 | 73.49% | 579 | 76.18% | 0.48 | 1.000 (referent) |  | 0.240 |
|  |  | CT | 185 | 24.28% | 166 | 21.84% |  | 1.161 (0.906-1.487) | 0.238 |  |
|  |  | CC | 17 | 2.23% | 15 | 1.97% |  | 1.186 (0.567-2.480) | 0.65 |  |
| DNAH5 | rs6554809 | CC | 541 | 71.18% | 593 | 78.03% | **0.006** | 1.000 (referent) |  | **0.007** |
|  |  | TC | 206 | 27.11% | 153 | 20.13% |  | **1.597 (1.246-2.048)** | **0.000** |  |
|  |  | TT | 13 | 1.71% | 14 | 1.84% |  | 1.235 (0.558-2.732) | 0.603 |  |
| PTGER4-DAB2 | rs7720838 | GG | 428 | 56.17% | 402 | 52.89% | 0.36 | 1.000 (referent) |  | 0.154 |
|  |  | GT | 291 | 38.19% | 306 | 40.26% |  | 0.957 (0.770-1.191) | 0.695 |  |
|  |  | TT | 43 | 5.64% | 52 | 6.84% |  | 0.837 (0.538-1.303) | 0.431 |  |
| SLC25A46-TSLP | rs10056340 | TT | 545 | 71.52% | 561 | 73.82% | 0.436 | 1.000 (referent) |  | 0.239 |
|  |  | GT | 202 | 26.51% | 189 | 24.87% |  | 1.093 (0.861-1.387) | 0.466 |  |
|  |  | GG | 15 | 1.97% | 10 | 1.32% |  | 1.293 (0.560-2.985) | 0.547 |  |
| TSLP | rs1898671 | CC | 695 | 91.21% | 693 | 91.18% | 0.901 | 1.000 (referent) |  | 0.944 |
|  |  | TC | 64 | 8.40% | 65 | 8.55% |  | 0.975 (0.671-1.417) | 0.896 |  |
|  |  | TT | 3 | 0.39% | 2 | 0.26% |  | 1.312 (0.216-7.953) | 0.768 |  |
| WDR36-CAMK4 | rs1438673 | TT | 221 | 29.31% | 250 | 33.03% | **0.029** | 1.000 (referent) |  | **0.014** |
|  |  | TC | 382 | 50.66% | 393 | 51.92% |  | 1.045 (0.824-1.324) | 0.718 |  |
|  |  | CC | 151 | 20.03% | 114 | 15.06% |  | **1.396 (1.021-1.908)** | **0.037** |  |
| SEMA6A-TNFAIP8 | rs6898653 | AA | 565 | 74.24% | 543 | 71.45% | 0.332 | 1.000 (referent) |  | 0.361 |
|  |  | GA | 174 | 22.86% | 198 | 26.05% |  | 0.840 (0.658-1.071) | 0.16 |  |
|  |  | GG | 22 | 2.89% | 19 | 2.50% |  | 1.098 (0.578-2.084) | 0.776 |  |
| RAD50 | rs6871536 | TT | 504 | 66.14% | 524 | 69.13% | 0.419 | 1.000 (referent) |  | 0.187 |
|  |  | CT | 233 | 30.58% | 214 | 28.23% |  | 1.312 (0.706-2.438) | 0.39 |  |
|  |  | CC | 25 | 3.28% | 20 | 2.64% |  | 1.132 (0.899-1.425) | 0.291 |  |
| PPM1A-DHRS7 | rs216518 | CC | 706 | 92.65% | 703 | 92.50% | 0.076 | 1.000 (referent) |  | 0.572 |
|  |  | CA | 56 | 7.35% | 52 | 6.84% |  | 1.063 (0.706-1.600) | 0.769 |  |
|  |  | AA | 0 | 0 | 5 | 0.66% |  | NA^c^ | NA^c^ |  |
| HLA-B-MICA | rs6932730 | TT | 552 | 73.21% | 551 | 73.27% | 1 | 1.000 (referent) |  | 0.982 |
|  |  | TC | 187 | 24.80% | 186 | 24.73% |  | 1.020 (0.799-1.302) | 0.872 |  |
|  |  | CC | 15 | 1.99% | 15 | 1.99% |  | 0.833 (0.396-1.752) | 0.63 |  |
| HLA-DQB1-HLA-DQB2 | rs7775228 | TT | 422 | 55.60% | 493 | 64.95% | **0.001** | 1.000 (referent) |  | **0.001** |
|  |  | TC | 275 | 36.23% | 216 | 28.46% |  | **1.589 (1.263-1.999)** | **0.000** |  |
|  |  | CC | 62 | 8.17% | 50 | 6.59% |  | 1.500 (0.996-2.259) | 0.052 |  |
| TWIST1-TMEM196 | rs7780001 | AA | 443 | 58.14% | 455 | 59.95% | 0.29 | 1.000 (referent) |  | 0.245 |
|  |  | TA | 276 | 36.22% | 274 | 36.10% |  | 0.997 (0.800-1.242) | 0.978 |  |
|  |  | TT | 43 | 5.64% | 30 | 3.95% |  | 1.575 (0.952-2.606) | 0.077 |  |
| NOD1 | rs7789045 | AA | 294 | 38.63% | 303 | 39.87% | 0.753 | 1.000 (referent) |  | 0.876 |
|  |  | TA | 355 | 46.65% | 340 | 44.74% |  | 1.063 (0.847-1.333) | 0.598 |  |
|  |  | TT | 112 | 14.72% | 117 | 15.39% |  | 1.049 (0.765-1.438) | 0.768 |  |
| GLI3 | rs4724100 | CC | 438 | 57.48% | 446 | 58.68% | 0.828 | 1.000 (referent) |  | 0.768 |
|  |  | CT | 278 | 36.48% | 266 | 35.00% |  | 1.060 (0.850-1.323) | 0.603 |  |
|  |  | TT | 46 | 6.04% | 48 | 6.32% |  | 0.952 (0.613-1.478) | 0.827 |  |
| CDHR3 | rs6967330 | GG | 653 | 85.70% | 669 | 88.03% | 0.4 | 1.000 (referent) |  | 0.177 |
|  |  | AG | 105 | 13.78% | 88 | 11.58% |  | 1.210 (0.884-1.657) | 0.235 |  |
|  |  | AA | 4 | 0.52% | 3 | 0.39% |  | 1.525 (0.310-7.510) | 0.604 |  |
| TPD52-ZBTB10 | rs6473223 | TT | 285 | 37.50% | 269 | 35.39% | 0.156 | 1.000 (referent) |  | 0.910 |
|  |  | CT | 353 | 46.45% | 388 | 51.05% |  | 0.861 (0.685-1.081) | 0.198 |  |
|  |  | CC | 122 | 16.05% | 103 | 13.55% |  | 1.077 (0.781-1.484) | 0.653 |  |
| PVT1 | rs4410871 | CC | 310 | 40.74% | 323 | 42.50% | 0.443 | 1.000 (referent) |  | 0.900 |
|  |  | TC | 364 | 47.83% | 340 | 44.74% |  | 1.142 (0.914-1.426) | 0.243 |  |
|  |  | TT | 87 | 11.43% | 97 | 12.76% |  | 0.927 (0.659-1.304) | 0.665 |  |
| IL33 | rs928413 | AA | 665 | 87.27% | 645 | 84.87% | 0.196 | 1.000 (referent) |  | 0.119 |
|  |  | GA | 95 | 12.47% | 109 | 14.34% |  | 0.875 (0.644-1.187) | 0.39 |  |
|  |  | GG | 2 | 0.26% | 6 | 0.79% |  | 0.421 (0.082-2.163) | 0.3 |  |
| GATA3-CELF2 | rs962993 | CC | 629 | 82.55% | 646 | 85.00% | 0.398 | 1.000 (referent) |  | 0.176 |
|  |  | CT | 127 | 16.67% | 110 | 14.47% |  | 1.160 (0.870-1.546) | 0.313 |  |
|  |  | TT | 6 | 0.79% | 4 | 0.53% |  | 2.027 (0.523-7.861) | 0.307 |  |
| C11orf30-LRRC32 | rs2155219 | TT | 234 | 30.75% | 227 | 29.87% | 0.673 | 1.000 (referent) |  | 0.455 |
|  |  | GT | 398 | 52.30% | 391 | 51.45% |  | 0.977 (0.770-1.240) | 0.849 |  |
|  |  | GG | 129 | 16.95% | 142 | 18.68% |  | 0.878 (0.644-1.197) | 0.411 |  |
| KIRREL3-ETS1 | rs10893845 | TT | 439 | 58.38% | 418 | 56.33% | 0.637 | 1.000 (referent) |  | 0.589 |
|  |  | GT | 262 | 34.84% | 276 | 37.20% |  | 0.910 (0.728-1.138) | 0.408 |  |
|  |  | GG | 51 | 6.78% | 48 | 6.47% |  | 1.052 (0.682-1.623) | 0.819 |  |
| STAT6 | rs1059513 | TT | 660 | 86.84% | 648 | 85.60% | 0.754 | 1.000 (referent) |  | 0.460 |
|  |  | CT | 97 | 12.76% | 105 | 13.87% |  | 0.902 (0.663-1.227) | 0.511 |  |
|  |  | CC | 3 | 0.39% | 4 | 0.53% |  | 0.491 (0.106-2.267) | 0.362 |  |
| TTC6 | rs1998359 | CC | 612 | 80.31% | 606 | 79.74% | 0.755 | 1.000 (referent) |  | 0.935 |
|  |  | CG | 136 | 17.85% | 143 | 18.82% |  | 0.953 (0.728-1.248) | 0.727 |  |
|  |  | GG | 14 | 1.84% | 11 | 1.45% |  | 1.228 (0.538-2.801) | 0.626 |  |
| SMAD3 | rs17228058 | AA | 732 | 96.19% | 725 | 95.52% | 0.513 | 1.000 (referent) |  | 0.513 |
|  |  | GA | 29 | 3.81% | 34 | 4.48% |  | 0.776 (0.461-1.307) | 0.34 |  |
|  |  | GG | 0 | 0.00% | 0 | 0.00% |  | NA^c^ | NA^c^ |  |
| C16orf72-GRIN2A | rs631208 | AA | 411 | 53.94% | 406 | 53.42% | 0.783 | 1.000 (referent) |  | 0.652 |
|  |  | GA | 298 | 39.11% | 294 | 38.68% |  | 1.011 (0.812-1.258) | 0.925 |  |
|  |  | GG | 53 | 6.96% | 60 | 7.89% |  | 0.870 (0.578-1.308) | 0.502 |  |
| CLEC16A | rs887864 | AA | 498 | 65.35% | 474 | 62.37% | 0.477 | 1.000 (referent) |  | 0.273 |
|  |  | GA | 229 | 30.05% | 249 | 32.76% |  | 0.874 (0.697-1.096) | 0.245 |  |
|  |  | GG | 35 | 4.59% | 37 | 4.87% |  | 0.775 (0.471-1.276) | 0.317 |  |
| CLEC16A | rs7203459 | TT | 633 | 83.07% | 604 | 79.58% | 0.177 | 1.000 (referent) |  | 0.123 |
|  |  | TC | 120 | 15.75% | 147 | 19.37% |  | **0.731 (0.555-0.962)** | **0.025** |  |
|  |  | CC | 9 | 1.18% | 8 | 1.05% |  | 0.962 (0.355-2.603) | 0.939 |  |
| IL21R -IL4R | rs2107357 | GG | 391 | 51.52% | 392 | 51.58% | 0.26 | 1.000 (referent) |  | 0.499 |
|  |  | AG | 304 | 40.05% | 320 | 42.11% |  | 0.920 (0.740-1.144) | 0.452 |  |
|  |  | AA | 64 | 8.43% | 48 | 6.32% |  | 1.191 (0.787-1.804) | 0.408 |  |
| GSDMB | rs2305480 | GG | 388 | 51.60% | 365 | 48.41% | 0.464 | 1.000 (referent) |  | 0.248 |
|  |  | GA | 302 | 40.16% | 322 | 42.71% |  | 0.918 (0.737-1.143) | 0.444 |  |
|  |  | AA | 62 | 8.24% | 67 | 8.89% |  | 0.884 (0.600-1.302) | 0.531 |  |
| ENTPD6 | rs1044573 | GG | 674 | 88.45% | 657 | 86.45% | 0.498 | 1.000 (referent) |  | 0.247 |
|  |  | GA | 83 | 10.89% | 97 | 12.76% |  | 0.795 (0.576-1.096) | 0.161 |  |
|  |  | AA | 5 | 0.66% | 6 | 0.79% |  | 0.768 (0.226-2.613) | 0.673 |  |
| NFATC2 | rs6021270 | TT | 674 | 88.45% | 683 | 89.87% | 0.54 | 1.000 (referent) |  | 0.318 |
|  |  | CT | 84 | 11.02% | 75 | 9.87% |  | 1.129 (0.803-1.586) | 0.485 |  |
|  |  | CC | 4 | 0.52% | 2 | 0.26% |  | 2.160 (0.374-12.472) | 0.389 |  |

a. Global *P* values [2 degrees of freedom (df)]: genotype frequencies in AR and control group were compared using a χ^2^ test with 2 df.

b. *P* values from unconditional logistic regression analyses, adjusted for age and gender.

c. NA, not available because of the rarity of genotype.

**Table S4.** Association analysis of 47 SNPs under dominant and recessive genetic model.

| Gene | SNP ID | Genetic model | Case | Control | logistic Regression | |
| --- | --- | --- | --- | --- | --- | --- |
|  |  |  |  |  | OR (95%CI) | *P*^a^ |
| PEX14 | rs2056417 | (AG+AA) vs.GG | 256/506 | 254/506 | 0.981 (0.787-1.222) | 0.861 |
|  |  | AAvs.(GG+AG) | 30/732 | 34/726 | 0.882 (0.526-1.478) | 0.633 |
| C1orf87-NFIA | rs12122228 | (GT+TT) vs.GG | 320/442 | 314/446 | 1.030 (0.834-1.271) | 0.784 |
|  |  | TTvs.(GG+GT) | 41/721 | 39/721 | 1.037 (0.650-1.655) | 0.878 |
| ABL2 | rs1325195 | (GA+AA) vs.GG | 564/198 | 564/196 | 0.999 (0.788-1.267) | 0.996 |
|  |  | AAvs.(GG+GA) | 199/563 | 186/574 | 1.127 (0.887-1.432) | 0.326 |
| RNF144A-ID2 | rs10174949 | (GA+AA) vs.GG | 312/450 | 343/417 | 0.860 (0.697-1.061) | 0.158 |
|  |  | AAvs.(GG+GA) | 49/713 | 57/703 | 0.865 (0.575-1.300) | 0.485 |
| IL1R1 | rs1558641 | (CT+TT) vs.CC | 321/431 | 296/449 | 1.133 (0.916-1.403) | 0.250 |
|  |  | TTvs.(CC+CT) | 29/723 | 36/709 | 0.735 (0.439-1.230) | 0.240 |
| IL1RL1-IL1RL2 | rs10189629 | (CA+AA) vs.CC | 117/645 | 126/634 | 0.861 (0.649-1.144) | 0.302 |
|  |  | AAvs.(CC+CA) | 8/754 | 4/756 | 1.485 (0.435-5.065) | 0.528 |
| IL1RL1 | rs3771175 | (AT+AA) vs.TT | 118/643 | 134/626 | 0.826 (0.624-1.092) | 0.180 |
|  |  | AAvs.(TT+AT) | 6/755 | 4/756 | 1.111 (0.306-4.031) | 0.873 |
| PLCL1 | rs10497813 | (GT+TT) vs.GG | 296/454 | 321/431 | 0.858 (0.693-1.061) | 0.158 |
|  |  | TTvs.(GG+GT) | 49/701 | 43/709 | 1.129 (0.731-1.744) | 0.586 |
| TMEM108 | rs7617456 | (GA+AA) vs.GG | 322/438 | 332/427 | 0.980 (0.794-1.209) | 0.849 |
|  |  | AAvs.(GG+GA) | 36/724 | 58/701 | **0.628 (0.403-0.980)** | **0.040** |
| LPP | rs9865818 | (GA+GG) vs.AA | 436/325 | 399/361 | 1.192 (0.967-1.469) | 0.100 |
|  |  | GGvs.(AA+GA) | 101/660 | 75/685 | 1.385 (0.999-1.922) | 0.051 |
| LPP | rs9860547 | (GA+AA) vs.GG | 487/274 | 461/299 | 1.138 (0.918-1.410) | 0.239 |
|  |  | AAvs.(GG+GA) | 131/630 | 106/654 | 1.275 (0.956-1.701) | 0.099 |
| DLG1-BDH1 | rs6583203 | (CT+CC) vs.TT | 50/712 | 55/705 | 0.845 (0.560-1.277) | 0.425 |
|  |  | CCvs.(TT+CT) | 0/762 | 0/760 | NA^b^ | NA^b^ |
| TLR1-TLR6 | rs2101521 | (AG+GG) vs.AA | 463/299 | 446/314 | 1.099 (0.889-1.358) | 0.384 |
|  |  | GGvs.(AA+AG) | 111/651 | 103/657 | 1.118 (0.829-1.509) | 0.464 |
| TLR1-TLR6 | rs17616434 | (TC+TT) vs.CC | 456/306 | 438/322 | 1.098 (0.889-1.357) | 0.384 |
|  |  | TTvs.(CC+TC) | 106/656 | 97/663 | 1.130 (0.832-1.534) | 0.435 |
| ADAD1 | rs17388568 | (GA+AA) vs.GG | 200/561 | 182/578 | 1.133 (0.891-1.440) | 0.307 |
|  |  | AAvs.(GG+GA) | 16/745 | 15/745 | 1.048 (0.497-2.207) | 0.902 |
| ADAD1-IL2 | rs17454584 | (GA+GG) vs.AA | 200/562 | 182/578 | 1.132 (0.890-1.439) | 0.312 |
|  |  | GGvs.(AA+GA) | 17/745 | 16/744 | 1.061 (0.516-2.184) | 0.872 |
| IL2 | rs2069772 | (CT+CC) vs.TT | 202/560 | 181/579 | 1.163 (0.915-1.478) | 0.217 |
|  |  | CCvs.(TT+CT) | 17/745 | 15/745 | 1.146 (0.550-2.390) | 0.716 |
| DNAH5 | rs6554809 | (TC+TT) vs.CC | 219/541 | 167/593 | **1.567 (1.231-1.995)** | **0.000** |
|  |  | TTvs.(CC+TC) | 13/747 | 14/746 | 1.098 (0.498-2.420) | 0.817 |
| PTGER4-DAB2 | rs7720838 | (GT+TT) vs.GG | 334/428 | 358/402 | 0.940 (0.763-1.159) | 0.563 |
|  |  | TTvs.(GG+GT) | 43/719 | 52/708 | 0.853 (0.553-1.314) | 0.470 |
| TMEM232-TSLP | rs10056340 | (GT+GG) vs.TT | 217/545 | 199/561 | 1.103 (0.874-1.393) | 0.410 |
|  |  | GGvs.(TT+GT) | 15/747 | 10/750 | 1.265 (0.550-2.912) | 0.580 |
| TSLP | rs1898671 | (TC+TT) vs.CC | 67/695 | 67/693 | 0.987 (0.684-1.424) | 0.943 |
|  |  | TTvs.(CC+TC) | 3/759 | 2/758 | 1.310 (0.216-7.931) | 0.769 |
| WDR36-CAMK4 | rs1438673 | (TC+CC) vs.TT | 533/221 | 507/250 | 1.124 (0.897-1.409) | 0.308 |
|  |  | CCvs.(TT+TC) | 151/603 | 114/643 | **1.359 (1.031-1.790)** | **0.029** |
| SEMA6A-TNFAIP8 | rs6898653 | (GA+GG) vs.AA | 196/565 | 217/543 | 0.863 (0.683-1.091) | 0.217 |
|  |  | GGvs.(AA+GA) | 22/739 | 19/741 | 1.147 (0.606-2.170) | 0.674 |
| RAD50 | rs6871536 | (CT+CC) vs.TT | 258/504 | 234/524 | 1.147 (0.919-1.434) | 0.226 |
|  |  | CCvs.(TT+CT) | 25/737 | 20/738 | 1.263 (0.683-2.338) | 0.457 |
| PPM1A-DHRS7 | rs216518 | (CA+AA) vs.CC | 56/706 | 57/703 | 0.987 (0.661-1.474) | 0.950 |
|  |  | AAvs.(CC+CA) | 0/762 | 5/755 | NA^b^ | NA^b^ |
| HLA-B-MICA | rs6932730 | (TC+CC) vs.TT | 202/552 | 201/551 | 1.005 (0.793-1.273) | 0.970 |
|  |  | CCvs.(TT+TC) | 15/739 | 15/737 | 0.829 (0.395-1.738) | 0.619 |
| HLA-DQB1-HLA-DQB2 | rs7775228 | (TC+CC) vs.TT | 337/422 | 266/493 | 1.571 (1.267-1.948) | **0.000** |
|  |  | CCvs.(TT+TC) | 62/697 | 50/709 | 1.276 (0.855-1.906) | 0.233 |
| TWIST1-TMEM196 | rs7780001 | (TA+TT) vs.AA | 319/443 | 304/455 | 1.050 (0.850-1.298) | 0.648 |
|  |  | TTvs.(AA+TA) | 43/719 | 30/729 | 1.577 (0.960-2.590) | 0.072 |
| NOD1 | rs7789045 | (TA+TT) vs.AA | 467/294 | 457/303 | 1.059 (0.856-1.311) | 0.598 |
|  |  | TTvs.(AA+TA) | 112/649 | 117/643 | 1.015 (0.758-1.358) | 0.922 |
| GLI3 | rs4724100 | (CT+TT) vs.CC | 324/438 | 314/446 | 1.043 (0.845-1.288) | 0.695 |
|  |  | TTvs.(CC+CT) | 46/716 | 48/712 | 0.930 (0.604-1.432) | 0.743 |
| CDHR3 | rs6967330 | (AG+AA) vs.GG | 109/653 | 91/669 | 1.220 (0.895-1.662) | 0.209 |
|  |  | AAvs.(GG+AG) | 4/758 | 3/757 | 1.487 (0.302-7.322) | 0.626 |
| TPD52-ZBTB10 | rs6473223 | (CT+CC) vs.TT | 475/285 | 491/269 | 0.907 (0.730-1.126) | 0.375 |
|  |  | CCvs.(TT+CT) | 122/638 | 103/657 | 1.173 (0.875-1.572) | 0.287 |
| PVT1 | rs4410871 | (TC+TT) vs.CC | 451/310 | 437/323 | 1.094 (0.886-1.351) | 0.405 |
|  |  | TTvs.(CC+TC) | 87/674 | 97/663 | 0.866 (0.629-1.193) | 0.380 |
| IL33 | rs928413 | (GA+GG) vs.AA | 97/665 | 115/645 | 0.854 (0.632-1.153) | 0.302 |
|  |  | GGvs.(AA+GA) | 2/760 | 6/754 | 0.428 (0.084-2.195) | 0.309 |
| GATA3-CELF2 | rs962993 | (CT+TT) vs.CC | 133/629 | 114/646 | 1.185 (0.893-1.572) | 0.239 |
|  |  | TTvs.(CC+CT) | 6/756 | 4/756 | 1.970 (0.510-7.607) | 0.325 |
| C11orf30-LRRC32 | rs2155219 | (GT+GG) vs.TT | 527/234 | 533/227 | 0.951 (0.758-1.192) | 0.660 |
|  |  | GGvs.(TT+GT) | 129/632 | 142/618 | 0.891 (0.679-1.169) | 0.403 |
| KIRREL3-ETS1 | rs10893845 | (GT+GG) vs.TT | 313/439 | 324/418 | 0.931 (0.753-1.151) | 0.510 |
|  |  | GGvs.(TT+GT) | 51/701 | 48/694 | 1.089 (0.712-1.666) | 0.694 |
| STAT6 | rs1059513 | (CT+CC) vs.TT | 100/660 | 109/648 | 0.883 (0.652-1.196) | 0.422 |
|  |  | CCvs.(TT+CT) | 3/757 | 4/753 | 0.498 (0.108-2.298) | 0.371 |
| TTC6 | rs1998359 | (CG+GG) vs.CC | 150/612 | 154/606 | 0.973 (0.751-1.262) | 0.839 |
|  |  | GGvs.(CC+CG) | 14/748 | 11/749 | 1.240 (0.545-2.823) | 0.608 |
| SMAD3 | rs17228058 | (GA+GG) vs.AA | 29/732 | 34/725 | 0.776 (0.461-1.307) | 0.340 |
|  |  | GGvs.(AA+GA) | 0/761 | 0/759 | NA^b^ | NA^b^ |
| C16orf72-GRIN2A | rs631208 | (GA+GG) vs.AA | 351/411 | 354/406 | 0.987 (0.801-1.215) | 0.899 |
|  |  | GGvs.(AA+GA) | 53/709 | 60/700 | 0.866 (0.582-1.288) | 0.478 |
| CLEC16A | rs887864 | (GA+GG) vs.AA | 264/498 | 286/474 | 0.861 (0.693-1.069) | 0.174 |
|  |  | GGvs.(AA+GA) | 35/727 | 37/723 | 0.811 (0.495-1.326) | 0.403 |
| CLEC16A | rs7203459 | (TC+CC) vs.TT | 129/633 | 155/604 | **0.743 (0.568-0.971)** | **0.030** |
|  |  | CCvs.(TT+TC) | 9/753 | 8/751 | 1.017 (0.377-2.748) | 0.973 |
| IL21R -IL4R | rs2107357 | (AG+AA) vs.GG | 368/391 | 368/392 | 0.956 (0.776-1.178) | 0.674 |
|  |  | AAvs.(GG+AG) | 64/695 | 48/712 | 1.236 (0.826-1.850) | 0.302 |
| GSDMB | rs2305480 | (GA+AA) vs.GG | 364/388 | 389/365 | 0.912 (0.740-1.124) | 0.387 |
|  |  | AAvs.(GG+GA) | 62/690 | 67/687 | 0.919 (0.632-1.336) | 0.657 |
| ENTPD6 | rs1044573 | (GA+AA) vs.GG | 88/674 | 103/657 | 0.793 (0.580-1.085) | 0.147 |
|  |  | AAvs.(GG+GA) | 5/757 | 6/754 | 0.791 (0.233-2.685) | 0.706 |
| NFATC2 | rs6021270 | (CT+CC) vs.TT | 88/674 | 77/683 | 1.155 (0.827-1.614) | 0.398 |
|  |  | CCvs.(TT+CT) | 4/758 | 2/758 | 2.134 (0.370-12.320) | 0.397 |

a. *P* values from unconditional logistic regression analyses, adjusted for age and gender.

b. NA, not available because of the rarity of genotype.

**Figure S1:** Meta-analysis results based on multiple gene lists by Metascape. The results of enrichment network visualized from the three gene lists (Bønnelykke, *et al.* (2013), Hinds, *et al.* (2013) and Ramasamy *et al.* (2011)). The associations within each study are represented as pie charts in the nodes and cluster labels were added manually. The enrichment network shows that cytokine production, regulation of cytokine secretion and positive regulation of interleukin-6 production are generally shared among all three lists. Th17 cell differentiation is shared between the Bønnelykke and Hinds lists.


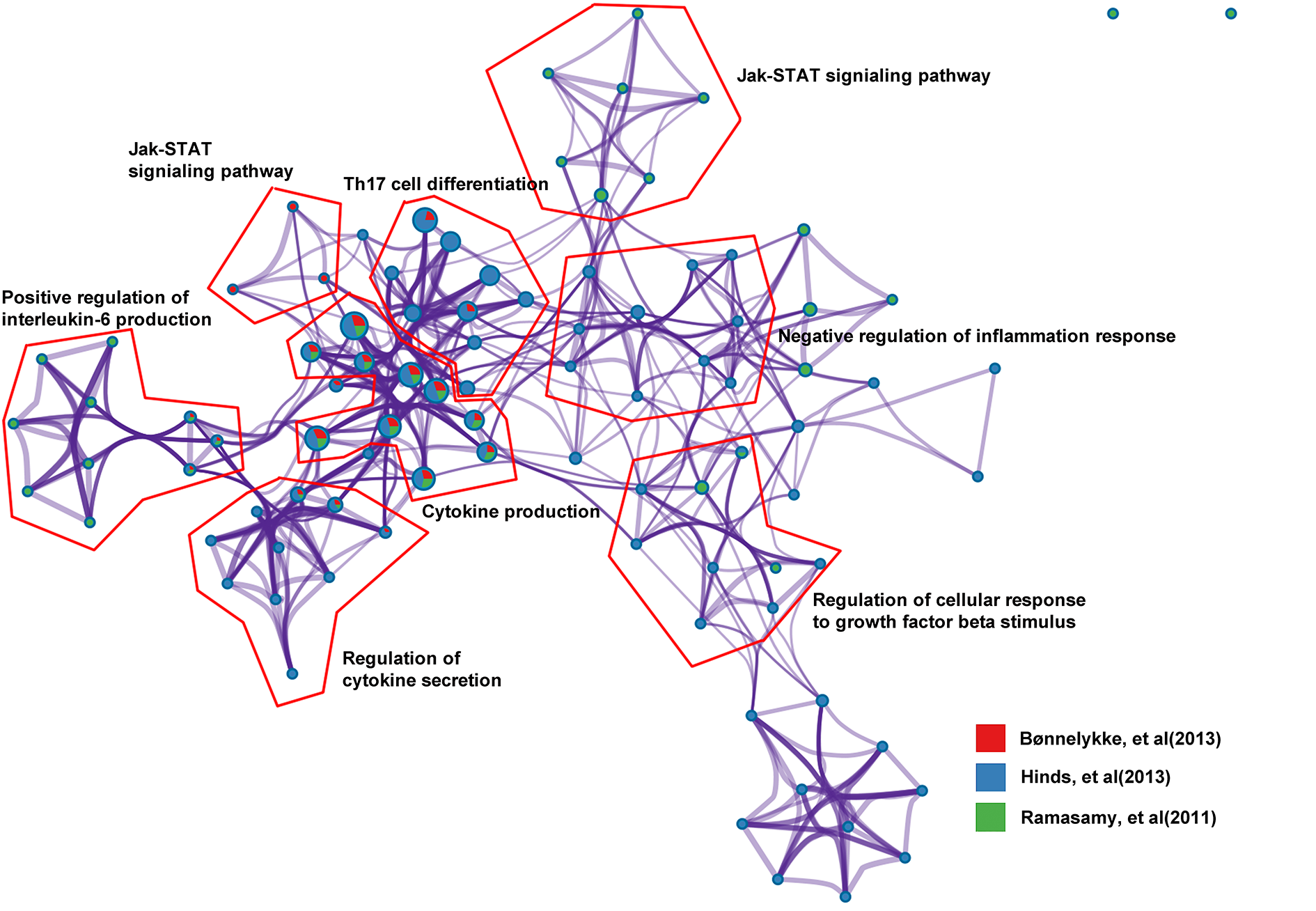


**Figure S2**.The flow diagram of recruitment subjects in AR group and control group


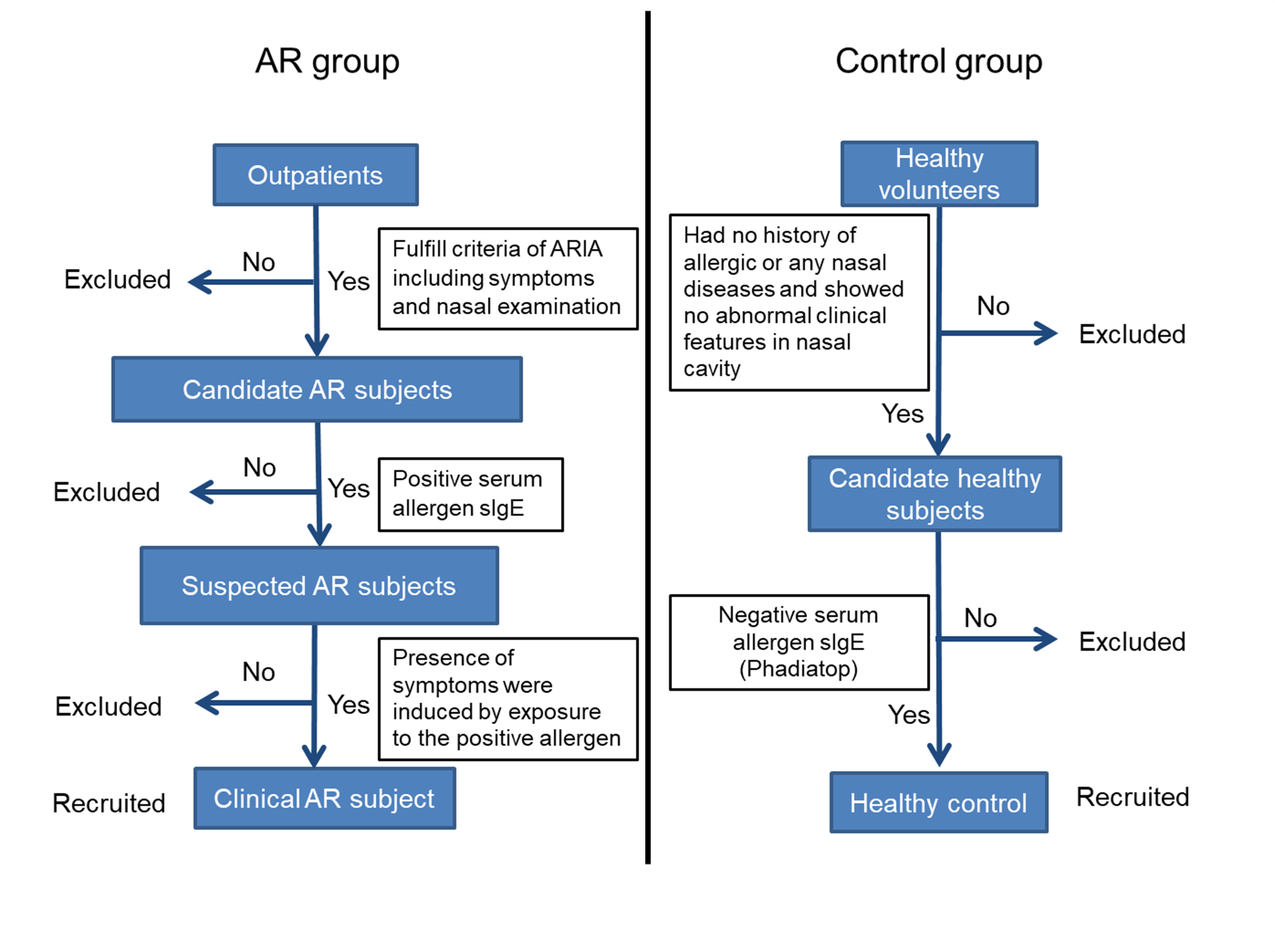


**Figure S3.** The association between genotypes and serum total IgE under recessive model, dominant model and codominant model analysis. (A) rs7617456 in TMEM108 gene. (B) rs6554809 in DNAH5 gene. (C) rs9865818 in LPP gene. (D) rs7203459 in CLEC16A gene. The level of serum IgE was described in median with interquartile range and Y axis was changed in logarithmic scale (lg= log_10_).


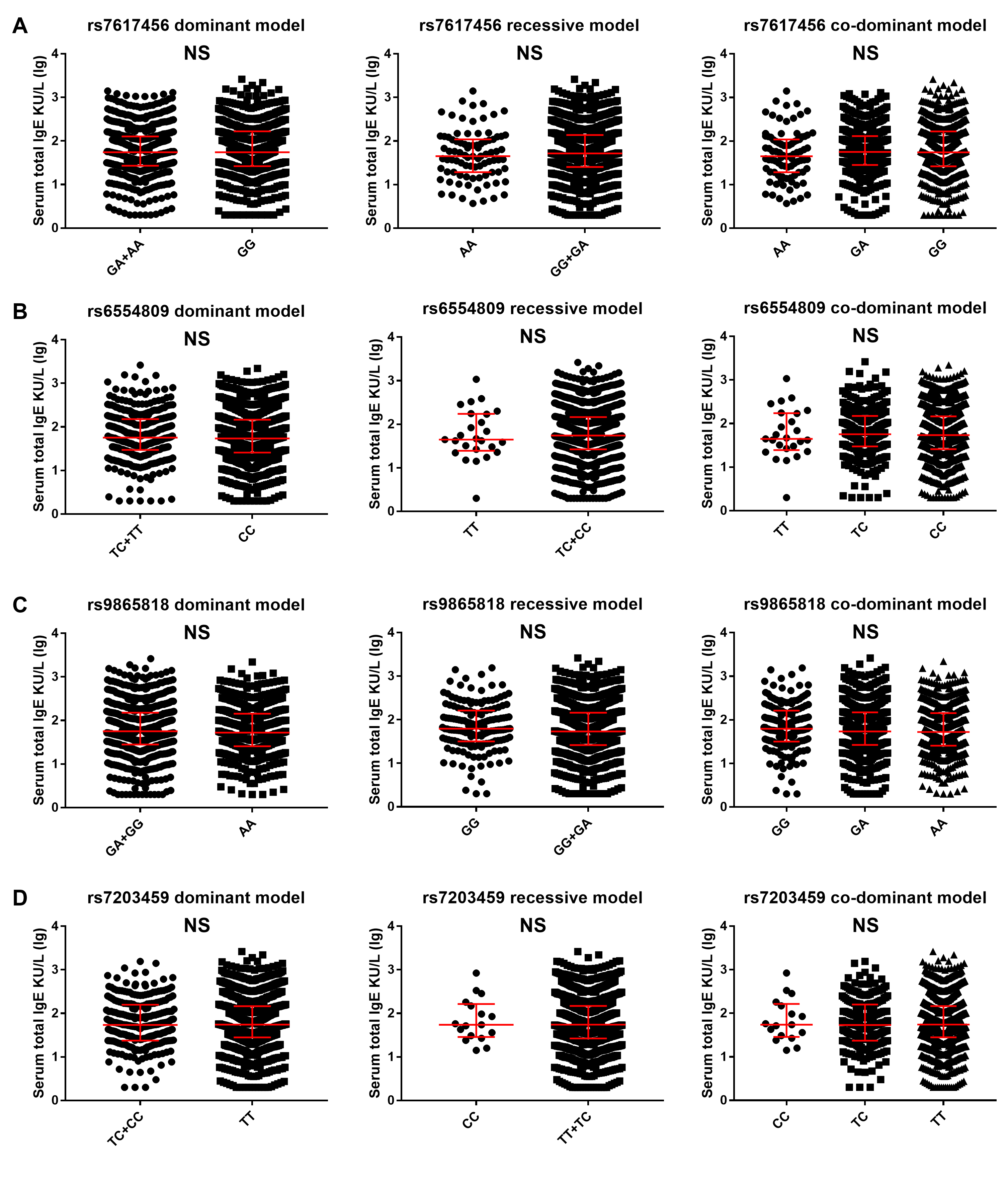

Supplement: Supplementary file 1 — Additional file 1. Additional tables and figures. [file 13223_2020_411_MOESM1_ESM.doc]
